# Identification of Nitrogen Fixation Genes in *Lactococcus* Isolated from Maize Using Population Genomics and Machine Learning

**DOI:** 10.3390/microorganisms8122043

**Published:** 2020-12-20

**Authors:** Shawn M. Higdon, Bihua C. Huang, Alan B. Bennett, Bart C. Weimer

**Affiliations:** 1 Department of Plant Sciences, University of California, Davis, CA 95616, USA; smhigdon@ucdavis.edu (S.M.H.); abbennett@ucdavis.edu (A.B.B.); 2 Department of Population Health and Reproduction, School of Veterinary Medicine, University of California, Davis, CA 95616, USA; bcahuang@ucdavis.edu; 3 100 K Pathogen Genome Project, University of California, Davis, CA 95616, USA

**Keywords:** *Lactococcus lactis*, biological nitrogen fixation, maize, pangenome, GWAS, random forests, lactococci, lactic acid bacteria, plant/microbe association

## Abstract

Sierra Mixe maize is a landrace variety from Oaxaca, Mexico, that utilizes nitrogen derived from the atmosphere via an undefined nitrogen fixation mechanism. The diazotrophic microbiota associated with the plant’s mucilaginous aerial root exudate composed of complex carbohydrates was previously identified and characterized by our group where we found 23 lactococci capable of biological nitrogen fixation (BNF) without containing any of the proposed essential genes for this trait (*nifHDKENB*). To determine the genes in *Lactococcus* associated with this phenotype, we selected 70 lactococci from the dairy industry that are not known to be diazotrophic to conduct a comparative population genomic analysis. This showed that the diazotrophic lactococcal genomes were distinctly different from the dairy isolates. Examining the pangenome followed by genome-wide association study and machine learning identified genes with the functions needed for BNF in the maize isolates that were absent from the dairy isolates. Many of the putative genes received an ‘unknown’ annotation, which led to the domain analysis of the 135 homologs. This revealed genes with molecular functions needed for BNF, including mucilage carbohydrate catabolism, glycan-mediated host adhesion, iron/siderophore utilization, and oxidation/reduction control. This is the first report of this pathway in this organism to underpin BNF. Consequently, we proposed a model needed for BNF in lactococci that plausibly accounts for BNF in the absence of the *nif* operon in this organism.

## 1. Introduction

*Lactococcus* is a genus of Gram (+) positive cocci that are widespread in applications of food biotechnology for fermentation [1,2,3] and more recently are being used as probiotic bacteria in animals and humans [4,5,6,7]. *Lactococcus lactis* is considered generally recognized as safe (GRAS) and critical for the production of fermented meat and dairy products. Often strain selection is based on their ability to produce lactic acid via sugar fermentation, capability to hydrolyze protein, and ability to synthesize polycyclic bacteriocins, such as Nisin. In combination, this provides a method of food preservation and flavor production in fermented foods [2,8,9,10,11]. Although humans are estimated to consume up to 10^18^ lactococcal cells per year from fermented dairy products [12], *L. lactis* is a minor but persistent resident of the human gastrointestinal tract, in part, because they survive gastrointestinal passage and become viable but non-culturable by relying on protein for energy [4,8,13,14,15,16,17,18]. Previous reports indicated that subspecies of *L. lactis* found to occur naturally in raw milk originated from numerous plant sources, including maize [8,19,20]. Phenotypic and molecular investigations provided evidence supporting a plant-based origin for domesticated *L. lactis* strains used in the dairy industry based on strong genetic similarities with plant-derived strains [21]. These findings were corroborated by comprehensive genomic comparisons of *L. lactis* isolates that revealed lactococci adapted for use in dairy fermentations exhibited a loss of functions likely necessary for survival in plant environments [22]. Moreover, recent studies demonstrated that strains of *L. lactis* are found in numerous plants as integral members of the microbiome and are likely to exhibit distinct genomes with uncharacterized metabolic capabilities [23,24,25,26].

The recent investigation of bacteria isolated from the aerial root mucilage of Sierra Mixe maize, a landrace variety that derives up to 82% of its nitrogen from the atmosphere, unexpectedly identified lactococci as diazotrophs that are capable of biological nitrogen fixation (BNF) [27]. Prior to these findings, diazotrophic lactococci were unreported. Interestingly, many lactococci isolated from Sierra Mixe maize exhibited the highest BNF phenotype among the >600 mucilage diazotrophs collected. However, the identification of the genetic underpinnings in the lactococcal genomes using hidden Markov models (HMMs) to search for well-known nitrogen fixation (*nif*) genes encoding components of the nitrogenase system revealed that the isolates completely lacked the minimum gene set proposed to be essential for BNF by Dos Santos et al., which includes the catalytic genes (*nifH*, *nifD*, and *nifK*) and genes involved with biosynthesis of the iron–molybdenum cofactor, FeMoCo (*nifE*, *nifN*, *nifB*) [28]. These results suggest that lactococci from Sierra Mixe maize utilize a novel mechanism for BNF and the prototypical metabolic pathway for the reduction of dinitrogen to ammonia via nitrogenase and the incorporation of atmospheric nitrogen into their metabolism was not found. This observation suggests that these lactococcal isolates use an unknown mechanism and pathway to fix nitrogen as well as the subsequent metabolism for incorporation and release to their plant host.

Advances in comparative microbial genomics methods provide a means to identify genes associated with these phenotypic results that will in part establish the mechanism for fixation via a new and novel route. The foundation for these analyses lies in the use of population genomics by constructing microbial pangenomes from whole genome sequencing (WGS) from closely related bacterial isolates with bioinformatic pipelines, such as Roary, which in turn opens the door to a range of downstream analyses that examine gene content and allelic variation to find putative genes underpinning the expressed phenotypes [29], including diazotrophy. For example, the resulting gene presence and absence information enables GWAS analysis in bacteria, where pangenome wide association studies (Pan-GWAS) with large populations of bacterial genomes serve as a method for the identification of genes associated with target phenotypes [30]. This Pan-GWAS approach was recently used to examine genetic deletions and successfully verified that only specific alleles of a core genome toxin gene (*porA*) caused abortion in livestock with *Campylobacter jejuni* infections with very high accuracy based on the population genomics approach [31]. While this strategy identifies genes of interest using widely accepted methods in statistical genetics, the implementation of machine learning algorithms to generate predictive models and identify genes with a ranked order of variable importance (VI) for targeted phenotypes is increasingly useful and provides an analytical method without a priori knowledge of the genes responsible for a trait [32]. In particular, decision tree-based models generated using random forests (RF) [33] can identify causal genes for phenotypes that include numerous forms of antimicrobial resistance and invasive diseases caused by human pathogens, such as *Escherichia coli*, *Salmonella enterica* and *Streptococcus pneumoniae* [34,35,36]. The successful use of these methods to identify the microbial genes associated with human and animal disease supports the use of this strategy to elucidate genomic features that are responsible for the ability of lactococci isolated from Sierra Mixe maize to conduct BNF without homologs to any essential, canonical *nif* genes. In this study, we hypothesized that the BNF phenotype in Sierra Mixe lactococci is attributed by novel genes that result in the cooperative fixation and release of atmospheric dinitrogen (N_2_) via genes that are unique to maize-associated isolates that will not be found in dairy-associated lactococci.

To test this hypothesis, we genomically examined 23 lactococcal isolates that have high BNF capacity from the aerial root mucilage of Sierra Mixe maize with 70 lactococcal isolates from dairy sources. Based on the positive BNF phenotypes of mucilage-derived lactococci [27] and reports of plant-associated lactococci displaying genomic heterogeneity from domesticated dairy strains [22], this larger set of dairy isolates served as a rational outgroup based on our hypothesis that lactococci from Sierra Mixe maize possess novel genes for BNF that are lacking in dairy isolates. Comparative genomic analysis confirmed that all maize-associated isolates were genomically unique *L. lactis* from dairy isolates and that lactococci derived from mucilage contained novel blocks of unique genomic features that were further examined for their potential role in BNF. Utilizing a combination of microbial Pan-GWAS and RF modeling, we identified a subset of genes associated with the BNF phenotype from the lactococcal pangenome that was only found in Sierra Mixe maize isolates. Many of the identified genes initially received hypothetical protein annotations, which led to exploiting the domain analysis as a means to provide functional insight for BNF association. This approach found genes with domains concomitant with utilization of Sierra Mixe maize mucilage polysaccharide derivatives, host-glycan adhesion, iron–siderophore utilization and BNF-related functions that are required for BNF in other characterized organisms. Finally, we integrated these results to propose a model for BNF in *L. lactis* that demonstrates the successful investigation of the hypothesis.

## 2. Materials and Methods

### 2.1. Bacterial Isolation, DNA Extraction and Whole Genome Sequencing

Isolation and WGS of *L. lactis* isolates from Sierra Mixe maize were recovered from plant samples using the culturing strategy and WGS methods previously described by Higdon et al. [27]. *L. lactis* isolates were obtained from the Weimer lab collection. They were accumulated over 25 years from various milk and dairy sources in the United States of America that primarily include hard cheeses but some from laboratory strains that were derived to be phage resistant [37]. DNA extraction and sequencing were done as previously described [38,39,40,41].

### 2.2. Whole Genome Assembly, Comparison and Taxonomic Analysis

Paired-end 150 FASTQ files from each isolate’s sequencing library were quality trimmed using Trimmomatic 0.36 with the following settings: ILLUMINACLIP:TruSeq3-PE.fa:2:40:15; LEADING: 2; TRAILING:2; SLIDINGWINDOW:4:15; MINLEN:50 [42]. Surviving read pairs were assembled using MEGAHIT 1.1.1 in paired-end mode using a default run parameters entailing a minimum multiplicity value of 2, a minimum k-mer size of 21, a maximum k-mer size of 141 and the iterative list of k-mer lengths that includes 21, 29, 39, 59, 79, 99, 119, 141 base pairs (bp) [43]. Genome assembly quality metrics were generated using QUAST 4.1 for each isolate genome assembly (Table S1) [44]. Fold coverage calculations for genome assemblies were achieved by mapping trimmed reads to their respective MEGAHIT assemblies using BWA 0.7.17 [45]. Computational analysis for the all-by-all whole genome comparison of the isolate genome collection was carried out using Sourmash 3.1.0 [46]. MinHash sketches used for the comparison of isolate genomes with Sourmash had an intermediate k-mer size of 31 with a scaled setting of 2000 in accordance with best practices for WGS comparisons of isolates from a single genus [47]. The comparative matrix output from Sourmash was visualized in R using the R package ComplexHeatmap 1.20.0 [48]. Taxonomic classifications of isolate MinHash sketches using the lowest common ancestor (lca) classify function of Sourmash 3.0.1 were done using MinHash sketches with a k-mer size of 31 and Genome Taxonomy Database v89 (available at: https://osf.io/gs29b/). Searches for the most similar *Lactococcus* reference genome to that of each BCW isolate (presented in Table S2) were conducted using the search function of Sourmash against a MinHash database of GenBank microbial genomes with a larger k-mer size of 51 to increase specificity (available at: https://osf.io/nemkw/) [49].

### 2.3. Pangenome Analysis

Protein coding features within the MEGAHIT genome assemblies of each isolate were identified and annotated using Prokka 1.12 [50]. Genomic feature files output from Prokka in GenBank format for each isolate were used as inputs for the pangenome analysis using Roary 3.12.0 [29]. Roary was run with the “-e” flag to generate a multi-FASTA alignment of core genes using PRANK and a minimum blastp identity value of 95%. Summary of the pangenome composition provided by Roary was visualized in Figure 2A using the open source python script ‘roary_plots.py’ hosted at: (https://github.com/sanger-pathogens/Roary/tree/master/contrib/roary_plots). Gene diversity estimation and visualization presented in Figure 2B was achieved using the native Rscript from Roary (*create_pan_genome_plots.R*). The visualization of the lactococcal pangenome presented in Figure 2C was achieved by uploading the gene presence and absence matrix output from Roary to the Phandango web server along with associated metadata for strain classification and isolation source [51]. The source code for the associated computational analysis and figure generation is available at: (https://github.com/shigdon/R-bcw-lac-analysis).

### 2.4. Identification of BNF Associated Genes by Pan-GWAS Analysis

The gene presence and absence matrix from Roary 3.12.0 constituting the pangenome for the lactococcal isolates of the BCW collections included in the study were input to Scoary 1.6.16 to carry out microbial pan-GWAS analysis. The 23 *L. lactis* genomes isolated from mucilage with previously described BNF phenotypes [27] were assigned a value of ‘1’ designating them as diazotrophic, and the 70 genomes of dairy isolates served as the non-diazotrophic control group that was assigned a value of ‘0’. Together, these 93 genomes comprised the dataset for pan-GWAS analysis in which the dairy control group was three times larger than the mucilage isolate group with confirmed BNF phenotypes. Genomic features from the pangenome were determined to be associated with the BNF phenotype upon surviving an adjusted *p*-value threshold (1 × 10^−7^) using the Benjamini–Hochberg method in Scoary. All genomic features surviving the filtering parameters were presented in Table S3. Additional filtering to identify subsets of genes based on classification groups (diazotroph vs. control) was performed in R using the dplyR package [52]. Shell scripts for the computational analysis are available at: (https://github.com/shigdon/bcw-lactococcus-shell).

### 2.5. Identification of Important Genes Using Random Forests

Decision tree modeling with the random forests (RF) algorithm was used to identify genes of high variable importance (VI) for the BNF phenotype, performed with R using the R package randomForest 4.6-14 [53]. To generate the RF classification model for the diazotrophic state, the gene presence and absence matrix from the *L. lactis* pangenome was paired with the data indicating binary state assignments of ‘diazotroph’ or ‘control’ (i.e., the out group). Each gene from the pangenome served as an independent variable utilized by the RF algorithm as a predictor. Settings for generation of the RF classification model included calculating proximity measurements among rows (genomes) included in the dataset and assessing the VI of each predictor (*P*). A 70–30 training/test split of input genome samples for the RF classification model was carried out using the R package rsample 0.0.7. The RF regression model was generated using the same R package and settings as the RF classification model. Rather than providing classification states, the data used for the regression model included a numeric vector of the previously determined BNF ratio [27] to represent the relative efficiency of the BNF phenotype for each diazotrophic *L. lactis* isolate. Values for *mtry* were determined using default settings of the randomForest 4.6-14 package, where *mtry* for the RF classification (RFC) model was defined as the square root of the number of predictors (9830 genes) and *mtry* for the RF regression (RFR) model was equal to *P*/3 (3267). Model accuracies were estimated using the R package Caret 6.0-84 [54] to apply the leave-out-out cross-validation (LOOCV) method. Each model was trained *n* times, where *n* was equal to the number of genomes and each iteration of *n* left out a different genome during model training. Genomic features were ranked in descending order based on the calculated values of VI and the subset using appropriate functions from the tidyverse 1.3.0 R package [55]. The intersections of identified genes determined to be important for (RF) or associated with (Pan-GWAS) the diazotrophic phenotype were visualized using the R package UpSetR 1.4.0 [56]. The source code used to generate each model, the associated metadata, data tables and gene intersection plots is provided on github at: (https://github.com/shigdon/rf-ms3).

### 2.6. Functional Annotation of Identified Protein Coding Sequences

Protein coding sequences that were determined to be statistically significantly associated with the BNF phenotype using Scoary 1.6.16 were subjected to comprehensive functional annotation beyond annotations provided by Prokka and Roary. Feature names reported from Scoary output were used to extract the corresponding nucleotide sequences from the pangenome reference file output by Roary. These gene sequences were gathered using the *filterbyname.sh* program from the BBtools package [57]. Each protein coding nucleotide sequence was queried against all of the default databases (TIGRFAM, SFLD, amap, SMART, CDD, ProSiteProfiles, ProSitePatterns, SUPERFAMILY, PRINTS, PANTHER, Gene3D, PIRSF, Pfam, Coils and MobiDBLite) provided by Interproscan 5.32-71 without a precalculated match lookup service (-dp), and with lookups enabled for Gene Ontology (-goterms) and InterPro (-iprlookup) annotations [58]. Shell commands used to implement Interproscan 5.32-71 are available at: (https://github.com/shigdon/bcw-lactococcus-shell). Raw output tables from Interproscan are available at: (https://github.com/shigdon/rf-ms3). Results obtained from Interproscan enabled the generation of the model presented in Figure 4 using Microsoft PowerPoint software.

### 2.7. Materials Availability

Genetic resources, including the biological materials and nucleic acid sequences from the maize-associated isolates, were accessed under an Access and Benefit Sharing (ABS) Agreement between the Sierra Mixe community and the Mars Corporation, and with authorization from the Mexican government. An internationally recognized certificate of compliance has been issued by the Mexican government under the Nagoya Protocol for such activities (ABSCH-IRCC-MX-207343-3). Any party seeking access to the nucleic acid sequences underlying the analysis reported here is subject to the full terms and obligations of the ABS agreement and the authorization from the government of Mexico. Individuals wishing to access nucleic acid sequence data for scientific research activities should contact Mars Incorporated Chief Science Officer at CSO@effem.com. None of the field sites were involved with endangered or protected species. The dairy lactococci WGS from the Weimer laboratory are available via NCBI Bioproject PRJNA186441.

## 3. Results

### 3.1. Comparison of WGS Assemblies Revealed Genomic Distance between Mucilage and Dairy Isolates

The lactococci isolated from dairy products were subjected to WGS and resulted in draft genome assemblies for use in comparative analysis with the *Lactococcus* WGS from Sierra Mixe maize, including their respective BNF ratio phenotypes [27]. The comparison of genome assembly metrics for the dairy isolate genomes with mucilage-derived lactococcal genomes were similar in the sequencing and assembly quality scores (Table S1). Complete taxonomic classification based on MinHash genome sketches using the LCA confirmed the assignment of each isolate to *Lactococcus lactis*. The subsequent strain level identification of each *L. lactis* genome revealed that the majority of dairy isolates were diverse and exhibited strong resemblance to 16 uniquely different strains (Table S2). These included two strains of *L. lactis* ssp. *lactis* bv. *diacetylactis*, six strains of *L. lactis* ssp. *cremoris*, seven strains of *L. lactis* ssp. *lactis*, and *L. lactis* strain Bpl1. The average genome similarity of dairy isolates to the nearest references in GenBank using MinHash whole genome sketches was 94%. The strain level classification of the 23 mucilage isolates revealed the nearest reference genomes corresponding to *L. lactis* ssp. *cremoris* KW2, five strains of *L. lactis* ssp. *lactis*, and *L. lactis* strain Bpl1. However, the average nucleotide similarity to GenBank *L. lactis* references for mucilage isolates was 56%. Interestingly, the most similar *L. lactis* reference genome in GenBank for BCW-000631 was also determined to be highly similar to *L. lactis* strain Bpl1, whereas BCW-000689 and BCW-000270 exhibited high similarity to the *L. lactis* ssp. *lactis* strain S0.

**Figure 1:**
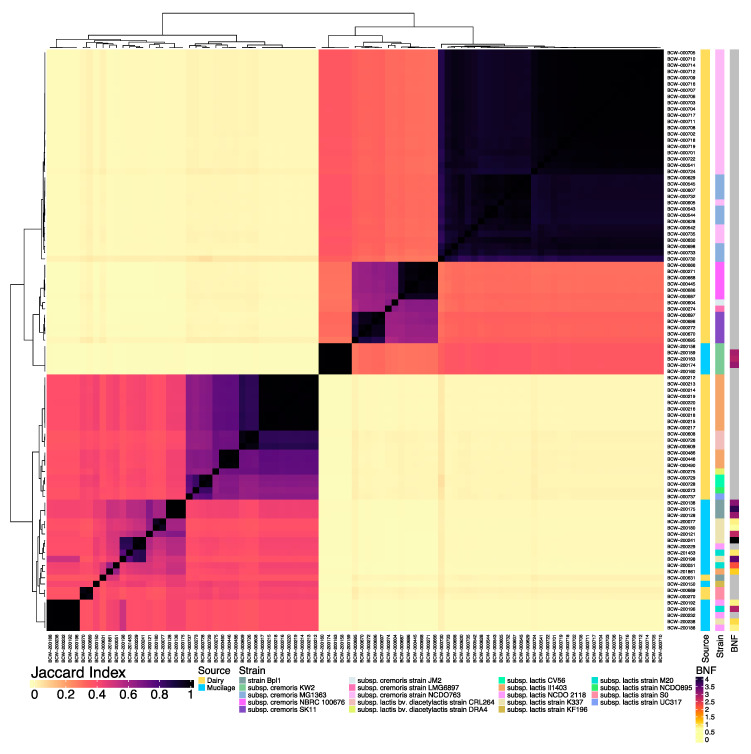
Whole genome sequencing (WGS) similarity of *L. lactis* isolates from dairy and mucilage. All-by-all comparison of the genome similarity generated using MinHash sketches from the draft genome assemblies for 23 lactococci isolated from Sierra Mixe mucilage and 70 *L. lactis* isolates from dairy isolates. MinHash sketches of each genome assembly used in the comparison were composed of k-mers with a length of 31 and a sketch size of 100,000. The Jaccard Index color bar represents the Jaccard Similarity Index (JSI) value computed for each pairwise comparison. JSI values of 1 indicate the total intersection of k-mers (Black) while JSI values of 0 correspond to highly dissimilar genome pairings (yellow). Isolation source annotations for each *L. lactis* isolate included dairy (dark yellow) and aerial root mucilage from Sierra Mixe maize (light blue). Color-coded annotations for strain classifications reflect the best matches for each genome to WGS references in GenBank (Table S2). Previously reported biological nitrogen fixation (BNF) ratios for mucilage-derived *L. lactis* isolates [27] were plotted alongside the comparative matrix. Light yellow coloration indicates BNF ratios close to 1, black shows the maximum observed value, and gray corresponds to isolates with no data available.

In spite of the genome diversity, all of the isolates were identified as lactococci so we proceeded with a direct comparison between the genomes from the two sources to identify unique genome content. Whole genome comparative analysis of isolates from these two sources revealed substantial variation in nucleotide composition and genome diversity. The all-by-all comparison of genome similarity among the 93 lactococcal genome assemblies revealed two distinct groups with isolates from each source in both major groups (Figure 1). However, dairy isolates with high similarity to *L. lactis* strain Bpl1 (BCW-000631) and *L. lactis* ssp. *lactis* strain S0 (BCW-000689 and BCW-000270) reference genomes in GenBank exhibited substantial genomic similarity to the largest cluster of mucilage lactococci. The evaluation of the BNF phenotypes observed for mucilage lactococci showed that the larger cluster of 18 isolate genomes had corresponding BNF ratios ranging from 0.9–4.1, while the smaller group of five diazotrophic lactococci exhibited BNF values of ~3. These results indicated the complexity of the BNF trait by highlighting the potential influence of genomic variation on BNF efficiency and suggested that some lactococci isolated from dairy sources may contain genes that participate in BNF reactions. As is commonly observed with lactococci, an enormous amount of strain variation was observed and often formed a unique genomic sub-structure within the large groups.

### 3.2. The Lactococcal Pangenome Revealed a Unique Genotype in Mucilage Isolates

**Figure 2:**
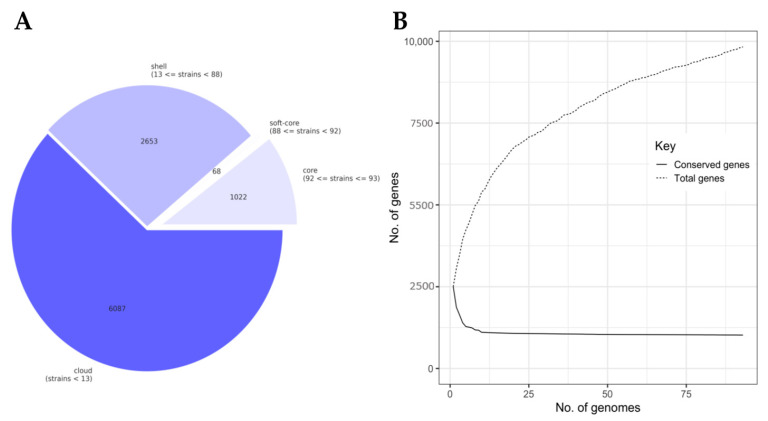

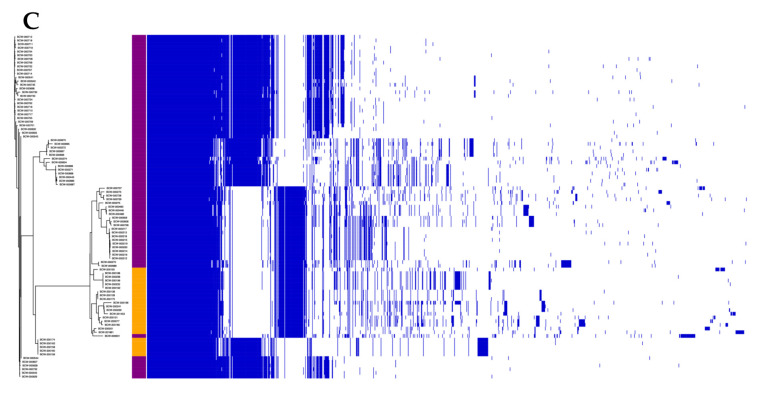
Pangenome analysis of *L. lactis* isolates from dairy and mucilage. Pangenome analysis of *L. lactis* isolate genomes was conducted using Roary [29]: (**A**) Summary of *Lactococcus* pangenome components based on individual genome feature presence; (**B**) Gene accumulation curve contrasting the number of total distinct, homologous genes (homologs) and conserved homologs comprising the *L. lactis* pangenome. Total genes represent the growth of the pangenome as newly identified genes were incorporated upon the stepwise addition of individual genomes. Conserved genes represent adjustment to the core genome size based on each isolate genome addition; (**C**) Matrix visualization of gene presence and absence output using Phandango [51] along with metadata indicating the isolation source of each genome. Orange annotations indicate genomes of *Lactococcus* isolates from Sierra Mixe mucilage and purple annotations designate those of lactococci isolated from dairy products.

Constructing a pangenome using draft assemblies of the 93 *L. lactis* isolates corroborated the genomic differences between the isolates from dairy and Sierra Mixe maize that were observed in the genome distance analysis. The total pangenome was comprised of 9830 homologous genes (homologs) with a core genome of 1022 conserved homologs that were present in 99–100% of all *L. lactis* isolates (Figure 2A). The soft-core genome was the smallest component of the pangenome and included homologs present over a range of 95–99% of lactococci genomes. The shell and cloud regions of the pangenome exhibited high degrees of genetic diversity with homolog counts of 2653 and 6087, respectively. Homologs comprising the shell-genome-populated isolate genomes to degrees ranging from 14 to 95%, while those assigned to the cloud-region were present in *L. lactis* genomes at levels of 14% or less. The examination of the gene accumulation curve (Figure 2B) revealed that the *L. lactis* pangenome exhibited rapid core genome stabilization within 15 genomes; however, additional genomes led to a high degree of genomic diversity for the constructed *L. lactis* pangenome and indicated that it was open [59].

Estimation of the genomic distance between *L. lactis* isolates using the accessory genome produced a phylogenomic tree consisting of five major clades (Figure 2C). The largest clade consisted of 41 genomes, of which 18 were derived from mucilage isolates and formed clusters independent from dairy isolate genomes with the exception of BCW-000631. Unlike the results from clustering genomes based on the whole genome distance (Figure 1), BCW-000689 and BCW-000270 formed an independent clade with a distinguished block of genes that was separated from the branch of the tree containing the majority of mucilage isolate genomes. In addition, an isolated group of five mucilage isolates comprised one of the major clades and exhibited a large block of unique genes, which corresponded to the observations made using the whole genome distance comparisons. In total, the pangenome analysis supported the hypothesis that *L. lactis* mucilage isolates are genomically distinct from those of dairy origins, yet some isolates for each source clustered together in both analyses.

### 3.3. Statistical and Machine Learning Analysis Identified L. lactis Genes Associated with BNF

Since the genotype was largely associated with the isolation source and the open genome indicated substantial gene variation, presumably due to the robust conjugation system in lactococci, we proceeded to determine the specific genes that may be associated with BNF. Utilizing the 70 *L. lactis* isolates from dairy as a control group, the Pan-GWAS analysis identified genes associated with the BNF phenotype observed in *L. lactis* isolates from Sierra Mixe maize. This statistical analysis of gene presence/absence data for each *L. lactis* genome utilized a reference matrix of phenotypic metadata that designated each isolate either as a diazotroph or member of the control group. This resulted in the identification of 214 unique clusters of homologs associated with the *L. lactis* BNF phenotype (Table S3). The resulting list included a mixture of gene sets that were either present exclusively in diazotrophic lactococci genomes, completely absent from the mucilage isolates, or found in both dairy and mucilage isolate genomes.

Filtering the identified homologs to identify the genes uniquely present in diazotrophic lactococci revealed a subset of 35 gene groups, for which 26 received hypothetical protein annotations and nine presented known functional annotations. Functional annotations in this set included homologs to the *bltD*, *btuD*, *prmC* and *xerD* genes with corresponding functions of spermine/spermidine acetyltransferase, vitamin B12 import, release factor glutamine methyltransferase, and tyrosine recombinase activities, respectively. However, the majority of functional genes coded for the uptake of iron from the environment as indicated by annotation, a unique capability that is not found in dairy lactococci as they do not rely on iron for dairy fermentation capabilities. Specifically, 16 *L. lactis* maize mucilage isolates possessed homologs to the *besA* and *yusV* genes corresponding to Ferri-bacillibactin esterase and an ATP-binding siderophore transport system, respectively. In addition, these isolates also possessed homologs for the *feuA*, *feuB*, and *feuC* genes encoding an iron–siderophore permease uptake system [60]. The analysis of the genetic structure for these five genes revealed a genomic arrangement in mucilage-derived lactococci as a single operon. The additional categorizing of the gene list to identify homologs that were completely absent from diazotroph genomes, yet present in dairy isolates, resulted in a subset of 103 unique groups of homologous genes. These genomic features included 42 different functional annotations as well as 59 groups that were also designated as hypothetical proteins (Table S3). These observations were consistent with our hypothesis and provided a context for metabolism associated with iron–sulfur cluster formation as the basis for canonical diazotrophy.

The information obtained by pangenome analysis was subsequently examined using the random forests (RF) algorithm to generate decision tree-based models to uniquely classify diazotrophic lactococci based on the genome content. Analogous to the approach taken with Pan-GWAS analysis, the 23 mucilage isolates received “diazotroph” state assignments while the 70 dairy isolates were defined as the non-diazotrophic “control” group. Passing these phenotypic assignments along with the 9830 genes from all 93 genomes from the pangenome as genetic variable inputs to the algorithm generated a preliminary RF classification (RFC) model that was used to identify genes of high variable importance (VI) for the BNF trait observed in *L. lactis* isolates from mucilage. The initial RFC model had an out-of-bag estimate of error rate (OOB-ER) of 1.08% and the estimate of model accuracy using the leave-one-out cross-validation (LOOCV) approach was 98.9%. These results provided a high degree of confidence in using our set of *L. lactis* WGS assemblies to generate a model capable of accurately predicting diazotrophic *L. lactis* isolates based on the WGS genotype. To test this and determine if genes of high VI identified by the initial RFC model were persistent, we designated the three dairy isolates previously found to exhibit high genomic similarity to mucilage isolates as a validation set (BCW-000270, BCW-000631, BCW-000689) and generated a second RFC model using a respective training/test split of 70 and 30% with 90 lactococcal genomes from both the diazotrophic and control groups. Similarly, the OOB-ER and estimated accuracy of the second RFC model were 0 and 100%, respectively. The comparison of the top 200 genes with high VI between the two RFC models revealed 84 common prediction variables (Figure S1). Interestingly, while the second RFC model predicted non-diazotrophic phenotypes for BCW-000270 and BCW-000689, it also predicted BCW-000631 to be diazotrophic based on its WGS genotype.

In addition to the RFC model, the previously reported BNF ratio values quantifying the relative diazotrophic efficiency of *L. lactis* isolates from mucilage served as phenotypic input to generate a RF regression (RFR) model that further supported the identification of genes with high VI for BNF [27]. Although generated using a specified number of decision trees identical to the RFC model, the regression model utilized an mtry value of 3276 (33% of the 9830 unique genes comprising the *L. lactis* pangenome used as prediction variables). This resulted in 43.1% of variance explained (R^2^) by the RFR model and a relatively low mean of squared residuals (RMSE) value of 0.58. Estimating the accuracy of these internal OOB-ER performance metrics by LOOCV showed similar R^2^ and RMSE values of 45.4% and 0.75, respectively. The subtle differences in the cross-validated performance metrics from the internal measurements based on OOB-ER provided additional confidence for the use of the RFR model to identify the genes associated with the BNF trait in *L. lactis*.

**Figure 3:**
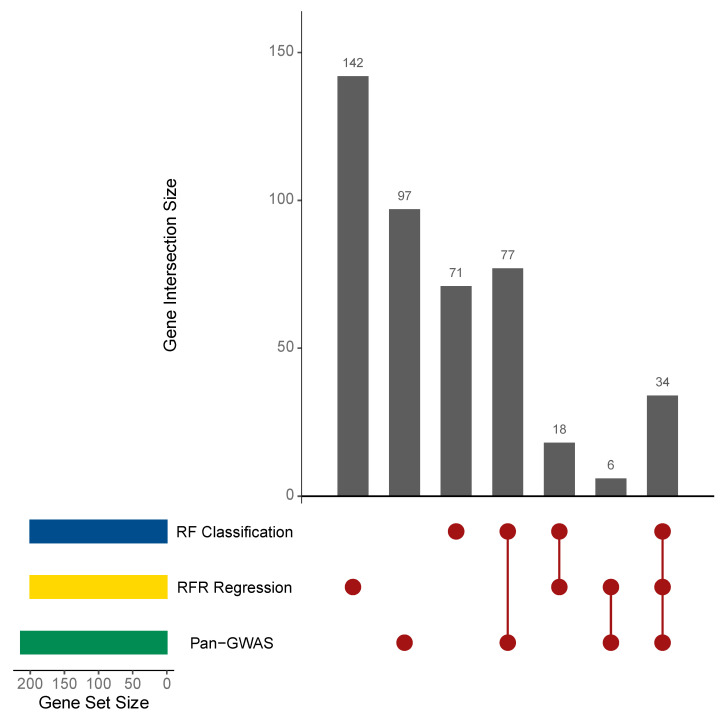
Comparison of the BNF-associated genes identified by Pan-GWAS and random forests. Genes associated with the BNF phenotype observed in *L. lactis* isolates from Sierra Mixe maize were identified using three different methods: microbial Pan-GWAS with Scoary [30], a random forests (RF) model of BNF state classification and a RF model regressing the observed BNF ratio values against gene presence and absence data for each isolate genome [32]. Numeric values indicate the number of homologous genes that were either uniquely identified by an implemented method, or common among two or more predictive approaches.

The comparison of the 214 homologs determined to be associated with the BNF trait by Pan-GWAS analysis with those of the highest VI identified using RF models demonstrated redundancy in gene identification among the implemented methods. Ranking prediction variables (homologs from the *L. lactis* pangenome) by individual contribution to the mean decrease in RFC model accuracy enabled the identification of the top 200 homologous genes with high VI for the BNF trait, of which 142 received hypothetical protein annotations. In a similar fashion, the 200 homologs from the RFR model with the highest degrees of VI—interpreted as those contributing the highest percentage increase in mean squared error—provided a suitable list for comparison and included 165 hypothetical protein annotations. Searching for commonality in the three lists revealed 34 homologs from the pangenome that were determined to be associated or important for the *L. lactis* BNF trait (Figure 3, Table 1). Direct comparison between the results from the Pan-GWAS and initial RFC model showed 111 homologs that were commonly identified, with 103 genes uniquely identified by Pan-GWAS and 89 exclusively determined by RFC. In sharp contrast, Pan-GWAS and the RFR model only shared 40 homologs predicted to be important for the BNF trait, and the majority of homologs determined to be important by RFR were distinguished from the other sets. Interestingly, the lists of variables with high VI identified by the RFC and RFR models demonstrated 52 shared homologs identified between them, constituting a commonality of ~25%. These results highlighted the benefits of combining Pan-GWAS and RF modeling to link genes with the BNF phenotype to identify the unknown genes associated with specific phenotypes and bolstered statistical support through feature identification redundancy for traits that have no known genetic basis.

### 3.4. Domain Annotations Elucidated Predicted Functions of Genes Associated with the BNF Trait

Since most of the genes associated with BNF were differentially contained between mucilage and dairy isolate genomes and annotated as hypothetical proteins, we conducted a comprehensive protein domain analysis of reference sequences from the pangenome with Interproscan [58]. This approach expanded the inferred biological functions of the 135 homologous gene groups commonly identified by Pan-GWAS and RF modeling as associated with BNF in *L. lactis* (Table S4). Using domain analysis led to the estimation of the protein function associated with BNF in *L. lactis* genes that received homology-based annotations to known genes, as well as for the majority of 76 gene groups with hypothetical protein annotations. Protein domain annotations of reference sequences for the 34 homologs identified as important to the *L. lactis* BNF trait by all three prediction methods revealed a diverse array of associated cellular and metabolic functions (Table 1). Domain annotations for seven of the homologs revealed functions related to membrane transport. These homologs consisted of two ATP-binding cassette (ABC) transporters associated with Vitamin B-12/Cobalamin (*btuD*) import, 2 transporters for D-xylose (*xylT*), an extracellular arabinose binding protein, an iron siderophore ABC transporter (*feuA*), and a citrate transporter (*ybiR*). Annotations of this subset also revealed homologs with functions pertaining to carbohydrate metabolism that included L-arabinose isomerase activity (*araA*), the hydrolysis of sucrose-6-phosphate (*scrB*), and a membrane-bound galactosyl transferase (GT) from family 1 (GT1). Regarding interactions between *L. lactis* and its extracellular environment, the domain annotation of this well supported subset indicated non-cytosolic proteins with functionalities conferring prokaryotic membrane lipoprotein lipid attachment and binding of the bacterial cell wall to the host surface. In addition, five of these commonly identified homologs received functional annotations for DNA binding factors, enzymes related to DNA modification, and transcription factors (TF) that are unique to maize isolates—all suggesting that the metabolic support machinery for diazotrophy in prototypical pathways are indicative of BNF in lactococci. However, the most intriguing discovery entailed the annotation of a hypothetical protein with predicted homology to the NifB protein that exhibits methyltransferase activity for the biosynthesis of the nitrogenase iron–molybdenum cofactor (FeMoCo) [61]. While these 34 genomic features received the highest level of support for association with BNF in *L. lactis*, the domain analysis of the 101 homologs that received dual (both RF methods) and the statistically supported confirmation of these important functions provided further insight to functions associated with the BNF trait in lactococci obtained from maize mucilage that are not common in dairy-associated isolates.

Domain annotations for the dually confirmed homologs of importance elucidated additional carbohydrate metabolism capabilities of diazotrophic *L. lactis* isolates. These included 15 homologs found to be present predominantly in mucilage lactococci genomes with an assortment of predicted functions (Table S4). Features related to carbohydrate transport uncovered in this subset included an ABC transporter with pentose specificity, as well as homologs for a phosphotransferase system (PTS) with predicted specificities for sucrose, lactose, cellobiose, and N,N′-diacetylchitobiose. Notable homolog annotations associated with pentose metabolism presented a ribulokinase (*araB*), L-ribulose-5-phosphate 4-epimerase (*araD*) and 1-deoxy-D-xylulose-5-phosphate synthase. The annotations also revealed homologs predicted to confer hydrolytic activities consisting of a peptidoglycan hydrolase/lysin, an alpha-1,2-mannosidase from the glycosyl hydrolase (GH) family 92, and a reducing-end xylose-releasing exo-oligoxylanase from GH family 8. Conversely, the identification of homologs associated with carbohydrate synthesis included genes encoding bacterial cellulose synthase subunit A (*bcsA*) and an enzyme from GT1 predicted in glycosylate serine-rich glycoproteins. Collectively, these features highlight the diversity of carbohydrate metabolism capabilities employed by maize-derived *L. lactis* isolates predicted to be positively associated with BNF.

Despite the detected presence of BNF-associated genes with carbohydrate-related functions in diazotrophic *L. lactis* genomes, a subset of homologs identified as important for the trait had predicted carbohydrate functionalities, yet lacked genomic presence in isolates with the BNF phenotype (Table S4). The majority of these homologs had functional annotations corresponding to the lactose metabolism. Specifically, *L. lactis* isolates from mucilage lacked homologs for the *lacB*, *lacC* and *lacD* genes conferring galactose-6-phosphate isomerase, tagatose-6-phosphate-kinase and tagatose 1,6-diphosphate aldolase activities, respectively. The absence of a homolog for the *lacF* gene encoding the IIA component of the lactose-specific PTS was also determined to be associated with the BNF trait. Carbohydrate-related genes of determined importance for BNF in *L. lactis* that were also absent from mucilage isolate genomes included homologs for glycerate-2-kinase (*garK*) and ribose-5-phosphate isomerase B (*rpiB*). The absence of these homologs from diazotrophic *L. lactis* genomes confirmed the differences in employed routes of carbohydrate catabolism and reflected differences between the environments from which mucilage- and dairy-associated strains were isolated. Lactose utilization is a trait widely recognized to be associated with adaptation to the milk environment that is distributed among dairy isolates via the *lac*-plasmid and conjugation [37]. Consequently, it is reassuring, and an internal validation, that lactose use is negatively associated with BNF.

Dually confirmed homologs for diazotrophic *L. lactis* isolates also had predicted functions for cell wall associated processes related to interactions of import for extracellular environment interaction. Among these was a coding sequence with a predicted homology to the *tagU* gene conferring teichoic acid attachment to peptidoglycan via phosphorylation-mediated transferase activity [62]. The domain analysis of several identified homologs with preliminary hypothetical protein annotations revealed functions related to situational host binding. These gene products had predicted functionalities that included a peptidoglycan-bound mucin-binding protein featuring a LPXTG motif, an extracellular galactose binding protein, and a predicted surface protein with domain homology for bacterial lectin activity. Other identified homologs that initially received hypothetical protein annotations presented functional domains consistent with a cell wall binding o-glycosyl hydrolase, a periplasmic solute binding protein featuring specificity for glyceraldehyde-3-phosphate (G3P), and an uncharacterized outer surface protein with different domains, respectively, conferring aldolase and peptidyl-prolyl isomerase activities. The identification of these activities demonstrated that the extracellular modification of compounds used to derive energy from binding and metabolizing sugars in the maize mucilage are likely an important function for diazotrophic lactococci. Furthermore, the annotation of these homologous protein coding sequences also presented two sequences exhibiting predictions for carbohydrate acetyltransferase activities that resembled a NodB-like chitooligosaccharide deacetylase and sialic acid synthase-related acetyl transferase, both of which are known functionalities associated with host–microbe interactions [63,64]. The detection of these homologs revealed that the genomes of diazotrophic *L. lactis* strains were enriched for novel genes that may confer necessary interactions with the surrounding environment to enable BNF activity via energy production from the complex carbohydrates available in mucilage [65] important to providing the energy needed for diazotrophy.

In addition to the identified activities related to carbohydrate transport, the domain annotation of important homologous genes for the BNF trait uncovered predicted proteins with functions related to the transport of metal ions and additional metabolites (Table S4). Increased genetic potential for iron utilization was reaffirmed by the repeated determination of homologs to *feuB*, *feuC* and *yusV* as important genes for diazotrophic lactococci, where their respective functional annotations denoted transmembrane ABC transporters facilitating the uptake of iron–siderophores—a function that is not found in dairy-associated lactococci. The dual identification of homologs predicted to encode membrane transporters with amino acid specificities also suggested that peptide utilization may be important for the BNF trait in *L. lactis*. These genes of determined importance consisted of homologs to the *tauB* gene known to encode an ABC transporter for the sulfonic amino acid taurine, and a hypothetical protein with domain annotations predicting permease activity for the translocation of amino acids and/or polyamines. Domain annotations for several homologs with hypothetical protein annotations suggested the importance for three additional transporters that included predicted activities resembling a cation–proton exchanger from the CPA1 family, a Metl-like transmembrane ABC transporter of unknown specificity, and a potentially novel transporter resembling those of the major facilitator superfamily (MFS). Collectively, the predicted substrate specificities of these transporters highlighted the potential differences in metabolic demands for iron- and sulfur-containing metabolites between mucilage lactococci and the strains isolated from dairy where the former group may exhibit different methods to control the oxidation and reduction potential to those found in dairy lactococci.

The examination of homologs identified as important for the BNF trait in *L. lactis* by Pan-GWAS and RFC uncovered coding sequences with predicted functionalities of potential significance to meeting known reductive and structural requirements of nitrogenase activity. Although the 93 *L. lactis* genomes investigated here lacked homologous sequences to the *nifD* and *nifK* genes conferring the oxidoreductase activity capable of reducing dinitrogen, Pan-GWAS and RFC analyses identified a novel homologous gene present in diazotrophic lactococci genomes with domain annotations indicating sequence similarity to an F420-dependent-like NADP oxidoreductase. These oxidoreductases are known to primarily exist in methanogenic archaebacteria, which utilize the F420 cofactor to reduce carbon dioxide for methane production [66]. In addition, the oxidoreductase activity carried out by dinitrogenase requires the assembly of the nitrogenase enzyme complex that is dependent on the biosynthesis of iron–sulfur clusters and thus, a sufficient supply of iron. Dual Pan-GWAS and RFC confirmation of homologs for the *besA* gene, as it is important for the BNF trait, along with its functional annotation predicting an esterase capable of liberating iron from imported siderophores, suggested that diazotrophic *L. lactis* isolates possessed the genomic means to meet the metabolic demands for iron required by BNF activity. Overall, these investigations found the biochemical components needed for diazotrophy without using the canonical genes from other organisms. Furthermore, these components were only found in maize-associated isolates and were largely absent in dairy-derived isolates.

## 4. Discussion

### 4.1. L. lactis Isolates from Mucilage Have Genomes Distinguished from Dairy Isolates

Surprisingly, lactococci obtained from Sierra Mixe maize mucilage were identified to have BNF activity [27]. To determine the underlying genetic capability for this phenotype, we conducted the comparative genomic analysis of these isolates with common organisms used in the dairy industry: the largest source of lactococci in food that are well characterized in terms of phenotype and genetic basis. The strategy to elucidate genes associated with the BNF phenotype relied upon the comparison of their genomes with those of a larger control group comprised of strains isolated from a dairy environment that were genomically distinct [27]. This strategy was based in part on previous reports of WGS comparisons with lactic acid bacteria (LAB) indicating that strains adapted to dairy environments originated from plant environments and experienced a loss of genes relevant for survival in plant niches [37,67]. The results from the comparison of the genome distance generated with *L. lactis* WGS assemblies corresponding to mucilage and dairy isolates corroborated the findings of a recent study reporting that the genomes of *L. lactis* strains adapted to dairy environments are distinguished from those associated with plants [68]. This led to the hypothesis that lactococci associated with the mucilage environment of Sierra Mixe maize possess genes that are conducive to the environmental requirements for BNF, but are uniquely different with respect to the *nif* gene content [27]. In response, we constructed a pangenome using all 93 *L. lactis* isolates and explored its contents using multiple computational approaches that included microbial Pan-GWAS and decision tree-based modeling with random forests prediction followed by a modified random forests method to validate the predictions and define the genes associated with BNF from the population of genes available in the open lactococcal pangenome. Working in concert, these computational steps revealed the distinct genomic character of *L. lactis* isolates from Sierra Mixe maize relative to that of dairy isolated strains, which uncovered a wide array of specially unique genomic features populating the cloud and shell regions of the *L. lactis* pangenome, and identified specific homologous genes associated with the BNF trait observed in mucilage-derived lactococci. Importantly, this approach also identified common genes in dairy lactococci that are negatively associated with BNF and served as an internal validation of the approach.

Measuring the relative genomic distance between all 93 isolates by comparing the genome-wide nucleotide composition of their WGS assemblies established a foundation to identify and characterize the distinguishing features of *L. lactis* isolates from mucilage. Results from the all-by-all comparison of WGS assemblies from mucilage and dairy isolates showed that plant-derived, diazotrophic genomes formed two independent clusters that were highly dissimilar from those of dairy isolates with the exception of three dairy genomes (Figure 1). This supported the body of reports indicating that dairy-adapted strains of *L. lactis* originated from plant-associated ancestors that exhibited broader metabolic capabilities lost to evolution over time [37,67,68]. Assessing the genomic distance between *Lactococcus* isolate genomes from mucilage and known *Lactococcus* reference genomes deposited in GenBank provided additional indications that mucilage lactococci were likely to exhibit novel genomic features based on the low levels of similarity in terms of the WGS nucleotide composition (Table S2). These reference genome comparisons revealed that the best-matching reference strains for mucilage isolates primarily consisted of *L. lactis* ssp. *lactis* strains, while the independent cluster of five mucilage isolates were closer to *L. lactis* ssp. *cremoris* KW2, likely because they contained the citrate utilization genes associated with diacetyl production used to name this subspecies. Although these results corroborated findings indicating that plant-derived *L. lactis* isolates have genotypes and phenotypes well aligned with ssp. *lactis* [68], the high levels of genome dissimilarity observed between mucilage lactococci and their nearest known relatives suggested their potential to confer novel metabolic functions yet to be described in maize-derived lactococci. However, the clustering of three dairy isolate genomes with the larger group of mucilage isolate genomes that exhibited a wide range of BNF efficiencies suggests that some *L. lactis* strains adapted to the dairy environment may have retained some genes that may confer BNF activity.

Constructing a pangenome using *L. lactis* isolates from dairy and mucilage served as a robust tool to comparatively visualize the biogeographical sources of the isolates as well as to identify and define the homologous genes associated with the BNF phenotype of mucilage lactococci. The large proportion of the pangenome populating its cloud region (Figure 2A), open nature of the pangenome upon completing the successive addition of all 93 isolate genomes (Figure 2B) and a high diversity of homologous gene distributions among *L. lactis* genomes (Figure 2C) conveyed the large degree of functional heterogeneity presented by isolates of *L. lactis* at the strain level. These results corroborate previous investigations showcasing how the open state of microbial pangenomes increases upon the addition of newly sequenced genomes from closely related taxonomic ranks from various sources, indicating that population genomics shift the taxonomical perspective of the metabolic capabilities of bacterial genera [69,70]. Additionally, the clustering of *L. lactis* genomes based on gene presence and absence (Figure 2C) unveiled the genomic features constituting the differences in whole genome nucleotide composition (Figure 1) observed between dairy and mucilage lactococci in the collection. These observations prompted the combination of microbial Pan-GWAS and RF machine learning methods to identify important homologous genes for the BNF trait.

While the use of Pan-GWAS to identify genes associated with observed phenotypes in bacteria is a widely accepted approach, the motivation for its co-implementation in this study alongside RF modeling stems from recent reports of its successful use to predict *L. lactis* phenotypes and their associated genomic determinants when provided with pangenome datasets [68,71]. The comparison of results from all three computational gene prediction methods (Figure 3) provided a prediction followed by a validation approach to strengthen the level of support for homologs identified as important to the BNF trait in *L. lactis* and also served to significantly expand the number of BNF-associated genetic targets. Although the initial RFC model generated using our complete (i.e., prediction) dataset classified the BCW-000631 isolate from dairy as diazotrophic, this observation was expected based on the genomic similarity of BCW-000631 to mucilage-derived *L. lactis* genomes (Figure 1 and Figure 2C) and was subsequently validated using the second RFC model. Additionally, comparing the 84 homologs found to be of high VI by both RFC models with the 34 high VI genes showcased in Table 1 revealed an intersection size of 26 genes. This result provided additional support in validating the RFC model generated here and suggested that it accurately predicts BNF phenotypes, and the underlying genes associated with this trait, for other strains of *L. lactis* subjected to similar pangenome analyses. Furthermore, functional domain analysis of homologs with predicted importance for the BNF trait provided biological context for genomic differences observed between mucilage and dairy lactococci.

### 4.2. Mucilage Lactococci Exhibit Genomic Potential to Create a Suitable Environment for BNF

**Figure 4:**
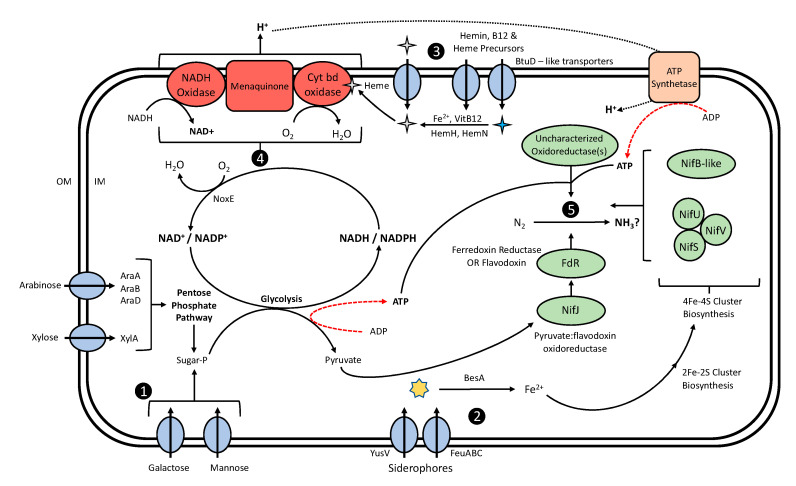
Proposed model for observed BNF activity in *L. lactis* associated with Sierra Mixe maize (**1**). Lactococci import monosaccharide derivatives of the aerial root mucilage polysaccharide comprised of pentoses (arabinose, xylose) and hexoses (galactose, mannose) [65,72] as their primary energy source for generating ATP and reducing equivalents based on their lack of a complete Krebs cycle [73]. Hexose sugars are phosphorylated and fed into glycolysis to generate ATP and reducing equivalents. Arabinose and xylose are catabolized via the non-oxidative branch of the pentose phosphate pathway (PPP) and fed into glycolysis as well through the activity of gene products from the *araABD* operon and the *xylA* gene, respectively; (**2**) Uptake of iron–siderophores (yellow star) is facilitated through action of the YusV and FeuABC transporters. Iron is liberated from siderophores by esterase activity of the BesA protein [60], generating cytosolic iron that is readily available for iron–sulfur cluster biosynthesis and other metabolic requirements; (**3**) The translocation of Hemin, Vitamin B-12 (cobalamin) and heme precursors (blue star) such as protoporphyrin IX occurs by the action of BtuD-like membrane transporters. Exogenous heme (gray star) uptake occurs through the action of ferrichrome ABC transporters [74,75], or heme assembly through the incorporation of cytosolic iron to imported heme precursors via ferrochelatase encoded by the *hemH* gene [76]; (**4**) Imported or synthesized heme compounds enable a membrane bound respiration chain (RC) and the subsequent elimination of molecular oxygen through the action of the cytochrome bd oxidase encoded by the *cydA* and *cydb* genes [77,78]. A membrane-bound NADH oxidase initiates electron transfer to menaquinone, which passes the reducing power to cytochrome bd oxidase for the reductive conversion of O_2_ to water and generates a proton motive force via H^+^ expulsion to the periplasm [78,79]. O_2_ depletion is also achieved through the action of cytosolic NADH oxidase. The resulting potential gradient may serve as an additional path for chemical energy by ATP-synthetase [79,80]; (**5**) Nitrogen fixation occurs through an uncharacterized system involving the coordination of NIF-related proteins and novel oxidoreductases conferring the reduction of dinitrogen to ammonia or an unknown metabolic product. The NifU, NifS and NifV proteins synthesize 4Fe–4S clusters, which interact with a novel NIFB-like protein to generate a FeMoCo-like metal cofactor potentially involved in the catalytic reduction. Pyruvate and ATP generated from sugar metabolism contribute to electron transfer between the NifJ protein and a secondary electron carrier (Ferredoxin Reductase or Flavodoxin), which relays the reducing equivalent to the site of catalysis.

The identification of genomic features associated with the BNF phenotype reported for *L. lactis* isolates from Sierra Mixe maize elucidated the components of the underlying mechanism conferring the trait. While the annotation of homologs comprising the constructed pangenome and subsequent functional domain analysis revealed genes conferring activities determined to be important for the BNF trait in *L. lactis* (Table 1, Tables S3 and S4), it is likely that many of the metabolic functions and biological processes inherent to homologous genes of the core and shell-genomes of the species contribute to the phenotype as well. We utilized the predicted functionalities of homologs identified as important for the diazotrophic phenotype along with knowledge of well supported metabolic processes affiliated with the species to generate a model that describes the means by which *L. lactis* associated with Sierra Mixe maize have the capacity to create an environment conducive to BNF (Figure 4).

#### 4.2.1. Mucilage Lactococci Exhibit the Potential to Utilize Mucilage Carbohydrates as Energy for BNF

BNF facilitated by the nitrogenase holoenzyme is known to be one of the most energy intensive biochemical transformations in biology, requiring the consumption of 16 ATP to reduce a single dinitrogen molecule to ammonia [81]. Due to the high stability of the triple bond constituting dinitrogen (N_2_) and previous confirmation that diazotrophic *L. lactis* genomes encode transporters for arabinose, galactose, mannose and xylose [27], we hypothesized that an analogous mechanism for diazotrophic *L. lactis* to reduce N_2_ would also impose heavy demands for ATP consumption; thereby, imposing an increase in genomic feature content related to the utilization of carbohydrates corresponding to the mucilage polysaccharide composition. Although lactococci adapted to dairy environments are known to utilize lactose for the generation of ATP [82], *L. lactis* isolates associated with plants express genes conferring the utilization of plant-based carbohydrates when cultured in the presence of plant leaf tissue lysate [83]. While plant-derived strains of *L. lactis* are reported to exhibit gene clusters conferring the ability to catabolize galactose for energy production [67], a large body of literature highlights the importance of their potential to derive ATP from arabinose and xylose catabolism [67,71,83,84].

Given that the diazotrophic *L. lactis* isolates investigated here originated from a plant host environment enriched by a mucilage polysaccharide containing both arabinosyl and xylosyl residues [65,72], the determined association of genes conferring the respective import and catabolism of these pentose sugars with the BNF phenotype suggested their importance for generating ATP and reducing equivalents (Table 1, Tables S3 and S4), both of which are required for prototypical diazotrophy. The utilization of arabinose as an energy source by *L. lactis* is a trait associated with plant-derived strains, yet uncommon in dairy production strains, such as IL1403 [85,86]. The identification of homologs for the *araA*, *araB*, *araD* genes as important for the BNF phenotype indicated that mucilage-associated lactococci are capable of degrading arabinose to generate D-xylulose-5-phosphate, feeding into the non-oxidative branch of the pentose phosphate pathway (PPP), in order to generate chemical energy and reducing power for BNF (Figure 4). Additionally, our analysis determined that an additional homolog for the *xylT* gene conferring xylose transport (xylT_2) was associated with the BNF trait, suggesting mucilage lactococci exhibit an increased demand for xylose uptake in their native environment (Table S4) that to date has not been found in dairy lactococci. This result corroborates an expression study of plant-derived *L. lactis* strains that reported the induction of the xylose isomerase gene (*xylA*) during cultivation on plant leaf tissue lysates, indicating an increase in metabolic flux towards non-oxidative PPP [83]. Additionally, the *rbsK*/*rpiB* gene encoding a bi-functional enzyme capable of either phosphorylating ribose, or reversibly converting it to ribulose 5-phosphate, was also determined to be associated with BNF in *L. lactis* (Table S3). When considered alongside the predicted importance for the presence of the *rbsA* gene, conferring ribose import and the absence of the unidirectional *rpiB* gene (Table S4), the genomic presence of the *rbsK*/*rpiB* homolog in diazotrophic *L. lactis* genomes suggests their potential to exhibit metabolic versatility with respect to the regulation of pentose catabolism and the generation of reducing equivalents for downstream biocatalytic processes, such as N_2_ reduction.

The Pan-GWAS and RF analyses also determined that the presence of homologs in diazotrophic *L. lactis* genomes with predicted functions involving mechanisms of host–microbe interactions were associated with the BNF trait (Table S4). The detection of the peptidoglycan-bound mucin-binding protein with the MucBP domain (group_8868) indicated mucilage lactococci are adapted to the mucilage environment and the sugars available within this structure. Interestingly, similar proteins have been reported in lactobacilli adapted to the human gastrointestinal tract environment [87]. In human systems, bacterial lectins are known to be adhesion proteins associated with the initiation of microbial attachment to host cells via the recognition of exposed host glycans [88]. The predicted importance of the hypothetical protein from homolog group 624 suggests that the ability of diazotrophic *L. lactis* to carry out a similar lectin-based mechanism for the attachment to glycans constituting the aerial root mucilage may be a significant functionality for their ability to fix nitrogen in the native environment. Additionally, teichoic acids attached to the cell walls of LAB are known to be involved in the colonization of the host and provide a reservoir of cations that may be essential for metabolic functions in *L. lactis* based on their anionic character [89]. This provides a biological context for the predicted association of homologs for the *tagU* gene (group_6282) with the *L. lactis* BNF phenotype, where the utilization of this gene by diazotrophic lactococci may provide them with a more effective means of host engagement and acquiring Fe^2+^ required for BNF. Furthermore, the predicted importance of genes conferring activities that enable diazotrophic lactococci to effectively adhere to the host surface draws relevance to the concomitant identification of the BNF-associated homolog (group_1288) with predicted NodB-like chitooligosaccharide deacetylase activity. The well supported involvement of this activity in mechanisms conferring symbiotic associations between rhizobium and leguminous plants suggests that an analogous signaling mechanism between diazotrophic *L. lactis* and its host may be important for the BNF trait [63,90].

The designation of genes related to glycan-adhesion as important for the BNF trait reinforces our results showing homologs for genes conferring GH activities are also important for diazotrophy in *L. lactis*. Although previous investigations into the carbohydrate utilization capabilities of diazotrophic *L. lactis* revealed their possession of homologs to a diverse array of GH genes with various monosaccharide specificities [27], Pan-GWAS and RF modeling identified BNF-association for GH homologs with predicted alpha-1,2-mannosidase and reducing-end xylose-releasing exo-oligoxylanase activities that correspond to GH families 92 and 8, respectively (Table S4). While the determined importance of these specific activities suggests that mucilage-associated *L. lactis* may utilize xylose and mannose to generate energy for BNF, the detection of these genes by prediction methods used here also corroborates a previous study reporting a high relative abundance of genes conferring these specific activities in the mucilage microbiome [91]. Taken together, the identification of these two GH homologs as important factors associated with the BNF phenotype supports the hypothesis that diazotrophic *L. lactis* derive energy for BNF from mucilage polysaccharide derivatives.

#### 4.2.2. Diazotrophic *L. lactis* Genomes Are Enriched with Iron Accumulation Genes

The assembly of the nitrogenase holoenzyme complex imposes a metabolic demand for iron on diazotrophic bacteria due to their need to synthesize iron–sulfur clusters [92,93]. We identified multiple genes with functional annotations related to the iron uptake from the extracellular environment that were determined to be important for the BNF trait in *L. lactis* isolates from mucilage. These included homologs for the genes of the *feuABC* operon and the *yusV* gene, which have both been characterized in *Bacillus subtilis* for their ability to confer iron–siderophore import from the extracellular environment [60,94]. Additionally, the presence and determined importance of a homolog for the *besA* gene conferring esterase activity provides these organisms with a sufficient mechanism to liberate cytosolic iron from siderophores [60]. Collectively, the confirmed presence of these homologs and predicted importance for the BNF trait provided evidence that diazotrophic strains of *L. lactis* may utilize homologs of the FeuABC and YusV transporters to effectively compete for iron–siderophores produced by other bacteria to meet the associated metabolic demand for iron (Figure 4).

#### 4.2.3. *L. lactis* Is Capable of Molecular O_2_ Depletion

A recently proposed model for nitrogen fixation in cereal crops suggested that plants, such as Sierra Mixe maize, may associate with free-living diazotrophs capable of generating O_2_ depleted environments conducive for BNF [95]. Due to their widespread application in fermentative industrial processes, species of microaerophilic *L. lactis* are well known for their ability to deplete oxygen, synthesize ATP, and achieve redox balance without the complete set of genes required for a fully functional TCA cycle [73]. Despite the absence of this electron transport chain system commonly found in respiratory bacteria, such as *Escherichia coli* and *Bacillus subtilis*, *L. lactis* is known to possesses the *cydA* and *cydB* genes required for the assembly of the cytochrome bd oxidase system that enables a functional respiration chain (RC) in the presence of oxygen and heme [78,96]. This observation is supported by numerous studies that have shown that *L. lactis* is capable of activating the cytochrome bd oxidase RC to produce O_2_ depleted bacterial environments, alleviate stress from reactive oxygen species via catalase, increase their long-term survivability, and potentially generate ATP via ATP-synthetase [74,76,77,79,80,97]. In addition to this membrane bound system, the cytosolic NADH oxidase encoded by *noxE* is known to be essential for *L. lactis* to lower the redox potential of milk cultures and deplete the concentration of molecular oxygen in the culture system [98]. Although heme is required for cytochrome bd oxidase activation and *L. lactis* lacks a complete heme biosynthetic pathway but is capable of accumulating heme via transport from the environment [78], maize is fully capable of synthesizing heme and may provide a reliable supply to the extracellular environment [99]. Coupling this with the demonstrated ability of *L. lactis* to acquire heme via import or synthesize it from protoporphyrin precursors and cytosolic iron with ferrochelatase [76] suggests that diazotrophic lactococci are very likely to possess the necessary genetic potential to reduce O_2_ levels through the utilization of the cytochrome bd oxidase system. This functionality may contribute to the low levels of molecular O_2_ observed in aerial root mucilage; thereby, enabling the associated BNF phenotype (Figure 4) [72]. Intriguingly, the investigation of the constructed *L. lactis* pangenome revealed that these genes are a part of the core genome, which explains their lack of determined importance for the BNF trait by the implemented prediction methods and supports previous proposals for their ubiquity among strains isolated from plant and dairy environments to control the redox potential that contributes to their microaerophilic lifestyle [78].

#### 4.2.4. Diazotrophic Lactococci Possess Genes with Domains Analogous to Nitrogenase Proteins

Previous investigation of *nif* gene presence in mucilage *L. lactis* genomes revealed homologous sequences for the *nifJ* gene involved in electron transfer for reduction of nitrogenase, and two proteins associated with 4Fe–4S cluster formation (*nif*S/*Isc*S—cysteine desulfurylase and *nifV*—homocitrate synthase) [27]. Species of *L. lactis* are also known to encode homologs for the *nifU* gene (*IscU*, *IscS*) associated with the assembly of Fe–S clusters [100,101]. These findings provide clear evidence that specific isolates of *L. lactis* possess the ability to transmit reducing power and synthesize Fe–S clusters, which are both known to be critical for function and assembly of the nitrogenase enzyme complex [61]. Additionally, the reaction carried out by the NifB protein involving the transfer of a methyl group from S-adenosyl-methionine (SAM) is known to be crucial for the synthesis of the nitrogenase metallocofactor [61]. The identification and highly supported association of pangenome homolog group 8186 with the BNF trait suggests that mucilage-associated *L. lactis* may be capable of synthesizing a metal cofactor analogous to that of diazotrophs possessing canonical *nif* gene operons through the activity of proteins with a NifB-like domain (Table 1). Further inspection of gene presence/absence results uncovered that dairy isolate BCW-000631 possessed this homolog while BCW-000270 and BCW-000689 did not, which corroborates the latter isolates designation as non-diazotrophic by our RFC model. Moreover, our computational approach identified a novel homolog comprising group 3929, which was found to be present only in diazotrophic *L. lactis* strains isolated from mucilage. The annotation of this gene determined to be important for the BNF trait by both Pan-GWAS and RFC modeling revealed that it encodes a protein with predicted activity similar to F420-dependent oxidoreductases utilized by methanogens to produce methane via CO_2_ reduction (Table S4) [66]. Interestingly, the ability of iron–molybdenum (MoFe) type nitrogenase to generate methane via CO_2_ reduction was recently characterized and confirmed [102]. This level of functional redundancy between F420-dependent oxidoreductases and MoFe-type nitrogenase suggests that while the novel oxidoreductase predicted to be important for BNF in *L. lactis* is likely to have specificity for CO_2_, it may also be able to reduce N_2_ to ammonia, as these proteins are often promiscuous for substrates.

## 5. Conclusions

*Lactococcus lactis* has been thoroughly investigated due to its use as a starter culture for the food biotechnology industry, especially in the dairy and fermented meat industries where the utilization of lactose to produce lactic acid has been a primary focus. Additionally, a recent proposal suggested LAB, including *L. lactis*, may be suitable agents for applications in agricultural biotechnology related to plant growth promotion [20]. Considering the confirmation of the lactococcal diazotrophic phenotypes in maize in spite of the demonstrated absence of *nifHDKENB* homologs [27] in these isolates, we hypothesized that *L. lactis* isolates from the mucilage microbiota of Sierra Mixe maize possess genes enabling BNF activity. Utilizing a group of 70 isolates from dairy as a control group to combine pangenome analysis with GWAS and machine learning methods, we identified genes with exclusive presence in the genomes of *L. lactis* isolates from mucilage that are predicted to be important for the BNF trait. The protein domain analysis of the identified unknown genes found in *L. lactis* isolates from mucilage that were largely lacking in the dairy isolates showed that molecular functions involving mucilage polysaccharide catabolism, glycan-mediated host adhesion, iron/siderophore utilization, FeMo cofactor biosynthesis (NifB), and novel oxidoreductase activities are important for the BNF trait. The integration of these results with information from additional research in the field led us to propose a model describing how mucilage-associated *L. lactis* may create a bacterial environment conducive to BNF. We elucidated that all the important genes for the BNF trait in *L. lactis* underpinning the mechanism for these isolates’ ability to fix atmospheric nitrogen exist in mucilage-derived lactococci, which supports the hypothesis that lactococci can be diazotrophs.

## Figures and Tables

**Table 1:** BNF-associated genomic features identified by pangenome wide association studies (Pan-GWAS), RF classification (RFC) and RF regression (RFR) models. Gene ID indicates the targeted cluster of homologous protein coding sequences in the *L. lactis* pangenome named using Prokka 1.12 and Roary 3.12.0 [35]. Interproscan annotations provide descriptions of the predicted function based on inference from multiple domain annotations provided by querying the identified sequences against all analyses available with Interproscan 5.32 [33]. The “*N* Mucilage” and “*N* Dairy” columns indicate the number of *L. lactis* isolate genomes of the respective isolation sources found to possess each homologous gene. Functional predictions for additional genes determined to be important for the BNF phenotype that were commonly identified by at least two of the computational analyses (Figure 3) are presented in Table S4.

Gene ID	Interproscan Annotation	*N* Mucilage	*N* Dairy
araA	L-arabinose isomerase	15	2
btuD_9	ABC Transporter Type 1	17	0
ddrA	Rad52/22 family double-strand break repair protein	0	64
feuA	Iron–siderophore periplasmic ABC transporter	16	0
group_1791	No match	17	0
group_1793	Putative *Lactococcus lactis* phage r1t holin	12	0
group_1931	Intrinsically disordered protein	0	60
group_2085	Glyoxalase/Bleomycin resistance/Dihydroxybiphenyl dioxygenase	23	3
group_2262	Intrinsically disordered protein	11	0
group_2959	MepB-like Protein of Unknown Function	14	0
group_2960	No match	11	0
group_3301	WxL domain surface cell wall-binding	6	68
group_3932	Putative DNA-binding domain superfamily	11	0
group_5428	Winged helix/Mga helix-turn-helix DNA binding	17	1
group_5436	Membrane-bound galactosyl-transferase/GT1	13	1
group_5483	No match	12	0
group_5487	Protein of unknown function DUF3892	12	0
group_5497	No match	12	0
group_5503	No match	11	0
group_5623	Type I DNA methyltransferase	11	0
group_5626	No match	11	0
group_6294	ROK (Repressor, ORF, Kinase) DNA-Binding Transcription Factor	18	3
group_696	No match	13	1
group_7770	Prokaryotic membrane lipoprotein lipid attachment	15	1
group_7798	Extracellular arabinose binding protein	13	1
group_7961	ABC Transporter Type 1	13	1
group_8186	FEMO cofactor biosynthesis protein NIFB	13	1
group_8190	RGG_Cterm: transcriptional activator, Rgg/GadR/MutR family	17	0
group_8270	No match	11	0
pspA	Histidine Phosphatase	23	3
scrB	Sucrose-6-phosphate hydrolase/glycosyl hydrolase family 32	17	5
xylT_1	SP: MFS transporter, sugar porter (SP) family	15	2
xylT_2	SP: MFS transporter, sugar porter (SP) family	20	3
ybiR	Citrate transporter	23	3

## Supplementary Materials

The following are available online at https://www.mdpi.com/2076-2607/8/12/2043/s1, Figure S1: Comparison of RFC model predictors of high variable importance, Table S1: Genome assembly quality metrics for *L. lactis* isolates, Table S2: Identification of nearest strain references for *Lactococcus* genomes, Table S3: BNF-associated genes identified by Pan-GWAS, Table S4: Annotation of BNF-associated genes commonly identified by Pan-GWAS and RF modeling.
