# Supplementary material for: Identification of Nitrogen Fixation Genes in Lactococcus Isolated from Maize Using Population Genomics and Machine Learning
Source: Microorganisms. 2020 Dec 20;8(12):2043. doi: 10.3390/microorganisms8122043 (PMC7768417; doi:10.3390/microorganisms8122043)
Supplement: Supplementary file 1 [file microorganisms-08-02043-s001.zip › Higdon_lactococcus_nif_SI_microorganisms.docx]

## Supplementary Materials

**Figure S1. Comparison of RFC model predictors of high variable importance.** The top 200 prediction variables (homologs from the *L. lactis* pangenome) ranked by highest variable importance were compared between the initial random forest classification (RFC) model – generated with all 93 *L. lactis* genomes – and the second RFC model trained in the absence of BCW-000270, BCW-00631 and BCW-000689 (dairy isolates that clustered with mucilage isolates). Gene identifiers for the 84 homologs constituting the intersecting group included: araA, araB, araR_1, bcsA, besA, btuD_9, ddrA, feuA, feuC_1, gatY, gmuE_1, group_1077, group_1288, group_1352, group_1660, group_1791, group_1810, group_1931, group_2066, group_2085, group_2262, group_2310, group_2588, group_2666, group_2667, group_2948, group_2959, group_298, group_3091, group_3096, group_3301, group_3903, group_3905, group_3929, group_3932, group_4133, group_4611, group_4754, group_4863, group_4996, group_5122, group_5428, group_5436, group_5497, group_5503, group_5623, group_5624 group_5625, group_5626, group_5776, group_5879, group_6074, group_6113, group_6282, group_6283, group_6284, group_6285, group_6294, group_729, group_7758, group_7770, group_7798, group_7961, group_8186, group_8190, group_8447, group_8868, group_8900, group_8907, group_8935, group_955, hsrA_1, kdpD, lacC_1, maa_1, nhaK, pspA, srrA, tauB, xerD_1, xylT_2, ybiR, ybjI_1, ybjI_2. See Table S3 for gene presence and absence information and Table S4 for annotation details for each of these homologous genes. The plot was generated using the UpSetR 1.4.0 package with R version 4.0.0 [1].

**Table S1. Genome assembly quality metrics for *L. lactis* isolates**

| **Isolate ID** | **Source** | **N contigs** | **Largest contig** | **Total length** | **GC (%)** | **N50** | **L50** | **Coverage** |
| --- | --- | --- | --- | --- | --- | --- | --- | --- |
| BCW-000212 | Dairy | 84 | 198606 | 2304378 | 35.11 | 82015 | 11 | 70 |
| BCW-000213 | Dairy | 80 | 198532 | 2302101 | 35.11 | 83747 | 10 | 42 |
| BCW-000214 | Dairy | 87 | 198616 | 2307390 | 35.11 | 74758 | 11 | 74 |
| BCW-000215 | Dairy | 81 | 232105 | 2302247 | 35.11 | 80307 | 10 | 54 |
| BCW-000216 | Dairy | 94 | 198616 | 2322354 | 35.1 | 74787 | 11 | 76 |
| BCW-000217 | Dairy | 88 | 198616 | 2305679 | 35.11 | 80307 | 11 | 46 |
| BCW-000218 | Dairy | 82 | 198617 | 2302945 | 35.11 | 80307 | 10 | 84 |
| BCW-000219 | Dairy | 98 | 198616 | 2350521 | 35.13 | 82855 | 10 | 106 |
| BCW-000220 | Dairy | 86 | 198616 | 2306555 | 35.11 | 74898 | 10 | 72 |
| BCW-000270 | Dairy | 77 | 429173 | 2516964 | 35.04 | 95777 | 7 | 109 |
| BCW-000271 | Dairy | 189 | 81824 | 2432098 | 35.51 | 24639 | 33 | 102 |
| BCW-000272 | Dairy | 175 | 154242 | 2469864 | 35.53 | 31559 | 24 | 82 |
| BCW-000273 | Dairy | 138 | 385517 | 2527777 | 34.97 | 71064 | 9 | 93 |
| BCW-000274 | Dairy | 217 | 129608 | 2380965 | 35.51 | 19605 | 36 | 119 |
| BCW-000275 | Dairy | 129 | 155425 | 2423612 | 34.98 | 75041 | 12 | 89 |
| BCW-000445 | Dairy | 216 | 67920 | 2431794 | 35.51 | 24638 | 33 | 74 |
| BCW-000446 | Dairy | 146 | 167744 | 2494491 | 34.98 | 48937 | 15 | 86 |
| BCW-000486 | Dairy | 154 | 147496 | 2455011 | 34.98 | 49642 | 16 | 306 |
| BCW-000490 | Dairy | 171 | 167704 | 2471357 | 35 | 49061 | 14 | 70 |
| BCW-000541 | Dairy | 124 | 395854 | 2530170 | 35.6 | 50388 | 12 | 111 |
| BCW-000542 | Dairy | 108 | 201044 | 2456035 | 35.58 | 64553 | 11 | 115 |
| BCW-000543 | Dairy | 82 | 265400 | 2328609 | 35.62 | 71304 | 10 | 56 |
| BCW-000544 | Dairy | 87 | 265400 | 2319302 | 35.61 | 73295 | 10 | 80 |
| BCW-000545 | Dairy | 98 | 201043 | 2346638 | 35.59 | 71716 | 12 | 268 |
| BCW-000604 | Dairy | 218 | 132572 | 2485283 | 35.46 | 29921 | 26 | 95 |
| BCW-000605 | Dairy | 111 | 270445 | 2383529 | 35.57 | 46126 | 12 | 134 |
| BCW-000607 | Dairy | 89 | 292663 | 2347798 | 35.58 | 73334 | 10 | 73 |
| BCW-000608 | Dairy | 171 | 135740 | 2588699 | 34.91 | 50493 | 15 | 96 |
| BCW-000609 | Dairy | 142 | 138066 | 2536513 | 34.91 | 58539 | 14 | 54 |
| BCW-000628 | Dairy | 97 | 235856 | 2353293 | 35.6 | 57378 | 12 | 37 |
| BCW-000629 | Dairy | 89 | 395857 | 2343856 | 35.59 | 73256 | 9 | 91 |
| BCW-000630 | Dairy | 105 | 292565 | 2390590 | 35.62 | 73278 | 10 | 65 |
| BCW-000631 | Dairy | 150 | 234973 | 2814653 | 34.73 | 86816 | 10 | 44 |
| BCW-000666 | Dairy | 191 | 67976 | 2391024 | 35.5 | 25660 | 33 | 70 |
| BCW-000668 | Dairy | 189 | 67920 | 2393815 | 35.5 | 25598 | 32 | 50 |
| BCW-000670 | Dairy | 177 | 154242 | 2472499 | 35.56 | 31046 | 25 | 62 |
| BCW-000686 | Dairy | 213 | 64254 | 2431659 | 35.52 | 24511 | 34 | 57 |
| BCW-000687 | Dairy | 250 | 67920 | 2424793 | 35.5 | 23210 | 35 | 401 |
| BCW-000689 | Dairy | 104 | 365568 | 2531169 | 35.04 | 82715 | 8 | 354 |
| BCW-000695 | Dairy | 365 | 131208 | 2608636 | 35.46 | 25562 | 30 | 217 |
| BCW-000696 | Dairy | 174 | 154243 | 2416858 | 35.53 | 31559 | 24 | 81 |
| BCW-000697 | Dairy | 166 | 149546 | 2405872 | 35.54 | 31559 | 24 | 75 |
| BCW-000698 | Dairy | 102 | 395857 | 2447178 | 35.55 | 56644 | 11 | 79 |
| BCW-000701 | Dairy | 121 | 334220 | 2490775 | 35.56 | 51876 | 12 | 79 |
| BCW-000702 | Dairy | 121 | 382878 | 2495170 | 35.58 | 58240 | 11 | 65 |
| BCW-000703 | Dairy | 119 | 382878 | 2494594 | 35.58 | 71390 | 10 | 88 |
| BCW-000704 | Dairy | 118 | 382878 | 2494191 | 35.58 | 50390 | 11 | 79 |
| BCW-000705 | Dairy | 127 | 169233 | 2496824 | 35.58 | 58248 | 13 | 127 |
| BCW-000706 | Dairy | 116 | 382878 | 2523144 | 35.59 | 51933 | 12 | 48 |
| BCW-000707 | Dairy | 116 | 382878 | 2494417 | 35.58 | 58166 | 11 | 65 |
| BCW-000708 | Dairy | 119 | 382878 | 2494554 | 35.58 | 52381 | 12 | 178 |
| BCW-000709 | Dairy | 146 | 382878 | 2481758 | 35.59 | 57211 | 11 | 162 |
| BCW-000710 | Dairy | 121 | 168226 | 2496765 | 35.57 | 58326 | 13 | 176 |
| BCW-000711 | Dairy | 121 | 265513 | 2496163 | 35.58 | 58248 | 12 | 229 |
| BCW-000712 | Dairy | 117 | 382878 | 2492026 | 35.58 | 58296 | 11 | 230 |
| BCW-000714 | Dairy | 124 | 214686 | 2495097 | 35.58 | 50388 | 12 | 146 |
| BCW-000716 | Dairy | 127 | 382878 | 2495396 | 35.58 | 57211 | 12 | 230 |
| BCW-000717 | Dairy | 122 | 382878 | 2495342 | 35.58 | 52381 | 11 | 222 |
| BCW-000718 | Dairy | 113 | 382879 | 2483006 | 35.56 | 62820 | 10 | 140 |
| BCW-000719 | Dairy | 114 | 265400 | 2482170 | 35.55 | 56546 | 12 | 117 |
| BCW-000722 | Dairy | 129 | 128350 | 2497066 | 35.57 | 50349 | 16 | 111 |
| BCW-000724 | Dairy | 156 | 382877 | 2514902 | 35.57 | 49592 | 11 | 394 |
| BCW-000726 | Dairy | 182 | 135640 | 2592804 | 34.9 | 51314 | 16 | 123 |
| BCW-000728 | Dairy | 128 | 162482 | 2508022 | 34.92 | 80425 | 11 | 105 |
| BCW-000729 | Dairy | 119 | 186323 | 2466625 | 34.95 | 85106 | 10 | 153 |
| BCW-000730 | Dairy | 250 | 192208 | 2559799 | 35.47 | 45798 | 15 | 264 |
| BCW-000732 | Dairy | 99 | 201043 | 2352568 | 35.6 | 57326 | 12 | 464 |
| BCW-000733 | Dairy | 115 | 214791 | 2444239 | 35.59 | 51833 | 13 | 384 |
| BCW-000735 | Dairy | 178 | 117940 | 2529665 | 35.58 | 45796 | 17 | 234 |
| BCW-000737 | Dairy | 154 | 151491 | 2509121 | 35.02 | 46599 | 17 | 195 |
| BCW-201861 | Mucilage | 47 | 385045 | 2416465 | 34.84 | 207796 | 4 | 192 |
| BCW-201453 | Mucilage | 36 | 301680 | 2528018 | 34.72 | 214273 | 5 | 162 |
| BCW-200051 | Mucilage | 26 | 479491 | 2373030 | 34.85 | 227838 | 4 | 333 |
| BCW-200077 | Mucilage | 47 | 477935 | 2610498 | 34.71 | 219555 | 4 | 48 |
| BCW-200121 | Mucilage | 40 | 450129 | 2482016 | 34.72 | 205251 | 4 | 212 |
| BCW-200128 | Mucilage | 26 | 484382 | 2341656 | 34.85 | 261159 | 3 | 194 |
| BCW-200138 | Mucilage | 28 | 483549 | 2341211 | 34.85 | 199730 | 4 | 255 |
| BCW-200150 | Mucilage | 27 | 405116 | 2458995 | 34.9 | 333528 | 4 | 177 |
| BCW-200158 | Mucilage | 25 | 540961 | 2358042 | 35.5 | 210530 | 4 | 253 |
| BCW-200159 | Mucilage | 24 | 515999 | 2356865 | 35.49 | 210530 | 4 | 274 |
| BCW-200160 | Mucilage | 22 | 540961 | 2357267 | 35.5 | 210530 | 4 | 253 |
| BCW-200163 | Mucilage | 25 | 540961 | 2357522 | 35.49 | 210530 | 4 | 259 |
| BCW-200174 | Mucilage | 26 | 540982 | 2354438 | 35.5 | 210530 | 4 | 300 |
| BCW-200175 | Mucilage | 28 | 454875 | 2341608 | 34.85 | 199730 | 4 | 310 |
| BCW-200180 | Mucilage | 47 | 466531 | 2617428 | 34.66 | 188527 | 5 | 153 |
| BCW-200188 | Mucilage | 31 | 272800 | 2453227 | 34.87 | 197140 | 6 | 103 |
| BCW-200192 | Mucilage | 27 | 272800 | 2452658 | 34.87 | 223913 | 5 | 264 |
| BCW-200196 | Mucilage | 26 | 272800 | 2452903 | 34.88 | 229112 | 5 | 324 |
| BCW-200198 | Mucilage | 269 | 451987 | 2874053 | 34.59 | 171646 | 6 | 294 |
| BCW-200229 | Mucilage | 67 | 451987 | 2490852 | 35.11 | 284906 | 4 | 290 |
| BCW-200232 | Mucilage | 26 | 272800 | 2452712 | 34.88 | 223913 | 5 | 247 |
| BCW-200238 | Mucilage | 28 | 272800 | 2452530 | 34.87 | 223913 | 5 | 230 |
| BCW-200241 | Mucilage | 28 | 451987 | 2463536 | 34.77 | 284924 | 4 | 200 |

Draft genome assemblies from MEGAhit [2] for each *Lactococcus* isolate were analyzed with Quast [3] to assess assembly quality. N contigs indicates the number of contiguous DNA sequences assembled with a length greater than 500 base pairs (bp). Numeric values for largest contig, total length, N50, and L50 were reported in bp. Coverage values reflect the average fold coverage contiguous sequences in each isolate genome assembly.

**Table S2. Identification of nearest strain references for *Lactococcus* genomes**

| **Isolate ID** | **Similarity** | **Accession** | **Name** | **Reference md5** |
| --- | --- | --- | --- | --- |
| BCW-000212 | 0.992 | AE005176.1 | Lactococcus lactis subsp. lactis Il1403 | 6490bef77c207ccdd87b638e1f7076d0 |
| BCW-000213 | 0.996 | AE005176.1 | Lactococcus lactis subsp. lactis Il1403 | 6490bef77c207ccdd87b638e1f7076d0 |
| BCW-000214 | 0.992 | AE005176.1 | Lactococcus lactis subsp. lactis Il1403 | 6490bef77c207ccdd87b638e1f7076d0 |
| BCW-000215 | 0.992 | AE005176.1 | Lactococcus lactis subsp. lactis Il1403 | 6490bef77c207ccdd87b638e1f7076d0 |
| BCW-000216 | 0.992 | AE005176.1 | Lactococcus lactis subsp. lactis Il1403 | 6490bef77c207ccdd87b638e1f7076d0 |
| BCW-000217 | 0.996 | AE005176.1 | Lactococcus lactis subsp. lactis Il1403 | 6490bef77c207ccdd87b638e1f7076d0 |
| BCW-000218 | 0.992 | AE005176.1 | Lactococcus lactis subsp. lactis Il1403 | 6490bef77c207ccdd87b638e1f7076d0 |
| BCW-000219 | 0.992 | AE005176.1 | Lactococcus lactis subsp. lactis Il1403 | 6490bef77c207ccdd87b638e1f7076d0 |
| BCW-000220 | 0.988 | AE005176.1 | Lactococcus lactis subsp. lactis Il1403 | 6490bef77c207ccdd87b638e1f7076d0 |
| BCW-000270 | 0.956 | CP010050.1 | Lactococcus lactis subsp. lactis strain S0 | e9c9abf5abf4dbed46ff925c0f8f38da |
| BCW-000271 | 0.791 | BCVK01000001.1 | Lactococcus lactis subsp. cremoris NBRC 100676 | 3c07cb57ac9ed68c7a8f54557ca25e79 |
| BCW-000272 | 0.996 | CP000425.1 | Lactococcus lactis subsp. cremoris SK11 | f7e619b941fbc5709d3e43e6ab4373a9 |
| BCW-000273 | 0.831 | LKLX01000084.1 | Lactococcus lactis subsp. lactis strain NCDO895 | 03124fa12a27f24e9451983b9f296a4b |
| BCW-000274 | 1.000 | LISZ01000065.1 | Lactococcus lactis subsp. cremoris strain LMG6897 | ef4da227bde6a3c79523ac4a70936164 |
| BCW-000275 | 0.910 | LIWD01000112.1 | Lactococcus lactis subsp. lactis bv. diacetylactis strain DRA4 | 6d4d4fd342d586bd8f7e709251313b60 |
| BCW-000445 | 0.784 | BCVK01000001.1 | Lactococcus lactis subsp. cremoris NBRC 100676 | 3c07cb57ac9ed68c7a8f54557ca25e79 |
| BCW-000446 | 0.753 | AE005176.1 | Lactococcus lactis subsp. lactis Il1403 | 6490bef77c207ccdd87b638e1f7076d0 |
| BCW-000486 | 0.753 | AE005176.1 | Lactococcus lactis subsp. lactis Il1403 | 6490bef77c207ccdd87b638e1f7076d0 |
| BCW-000490 | 0.743 | AE005176.1 | Lactococcus lactis subsp. lactis Il1403 | 6490bef77c207ccdd87b638e1f7076d0 |
| BCW-000541 | 0.975 | LITG01000066.1 | Lactococcus lactis subsp. cremoris strain NCDO763 | 0da443d01ac9c87a6d3dbdf56d16d5b7 |
| BCW-000542 | 0.935 | LITG01000066.1 | Lactococcus lactis subsp. cremoris strain NCDO763 | 0da443d01ac9c87a6d3dbdf56d16d5b7 |
| BCW-000543 | 0.954 | AM406671.1 | Lactococcus lactis subsp. cremoris MG1363 | d859bb5f7c0a26e636d9df3629db0dfc |
| BCW-000544 | 0.950 | AM406671.1 | Lactococcus lactis subsp. cremoris MG1363 | d859bb5f7c0a26e636d9df3629db0dfc |
| BCW-000545 | 0.950 | AM406671.1 | Lactococcus lactis subsp. cremoris MG1363 | d859bb5f7c0a26e636d9df3629db0dfc |
| BCW-000604 | 0.943 | CP015900.1 | Lactococcus lactis subsp. cremoris strain JM2 | 83f4c7fd2bb3e93218f8b05323a20cec |
| BCW-000605 | 0.927 | LITG01000066.1 | Lactococcus lactis subsp. cremoris strain NCDO763 | 0da443d01ac9c87a6d3dbdf56d16d5b7 |
| BCW-000607 | 0.950 | AM406671.1 | Lactococcus lactis subsp. cremoris MG1363 | d859bb5f7c0a26e636d9df3629db0dfc |
| BCW-000608 | 0.952 | LKPE01000001.1 | Lactococcus lactis subsp. lactis bv. diacetylactis strain CRL264 | 52df96765200788ed5ffc1d71d8d04a9 |
| BCW-000609 | 0.938 | LKPE01000001.1 | Lactococcus lactis subsp. lactis bv. diacetylactis strain CRL264 | 52df96765200788ed5ffc1d71d8d04a9 |
| BCW-000628 | 0.934 | AM406671.1 | Lactococcus lactis subsp. cremoris MG1363 | d859bb5f7c0a26e636d9df3629db0dfc |
| BCW-000629 | 0.950 | AM406671.1 | Lactococcus lactis subsp. cremoris MG1363 | d859bb5f7c0a26e636d9df3629db0dfc |
| BCW-000630 | 0.954 | LITG01000066.1 | Lactococcus lactis subsp. cremoris strain NCDO763 | 0da443d01ac9c87a6d3dbdf56d16d5b7 |
| BCW-000631 | 0.643 | JRFX01000001.1 | Lactococcus lactis strain Bpl1 | 8bb572e8e35407522ac8e8f9ce288b04 |
| BCW-000666 | 0.784 | BCVK01000001.1 | Lactococcus lactis subsp. cremoris NBRC 100676 | 3c07cb57ac9ed68c7a8f54557ca25e79 |
| BCW-000668 | 0.791 | BCVK01000001.1 | Lactococcus lactis subsp. cremoris NBRC 100676 | 3c07cb57ac9ed68c7a8f54557ca25e79 |
| BCW-000670 | 0.991 | CP000425.1 | Lactococcus lactis subsp. cremoris SK11 | f7e619b941fbc5709d3e43e6ab4373a9 |
| BCW-000686 | 0.788 | BCVK01000001.1 | Lactococcus lactis subsp. cremoris NBRC 100676 | 3c07cb57ac9ed68c7a8f54557ca25e79 |
| BCW-000687 | 0.761 | BCVK01000001.1 | Lactococcus lactis subsp. cremoris NBRC 100676 | 3c07cb57ac9ed68c7a8f54557ca25e79 |
| BCW-000689 | 0.956 | CP010050.1 | Lactococcus lactis subsp. lactis strain S0 | e9c9abf5abf4dbed46ff925c0f8f38da |
| BCW-000695 | 0.900 | CP000425.1 | Lactococcus lactis subsp. cremoris SK11 | f7e619b941fbc5709d3e43e6ab4373a9 |
| BCW-000696 | 0.969 | CP000425.1 | Lactococcus lactis subsp. cremoris SK11 | f7e619b941fbc5709d3e43e6ab4373a9 |
| BCW-000697 | 0.965 | CP000425.1 | Lactococcus lactis subsp. cremoris SK11 | f7e619b941fbc5709d3e43e6ab4373a9 |
| BCW-000698 | 0.992 | AM406671.1 | Lactococcus lactis subsp. cremoris MG1363 | d859bb5f7c0a26e636d9df3629db0dfc |
| BCW-000701 | 0.987 | LITG01000066.1 | Lactococcus lactis subsp. cremoris strain NCDO763 | 0da443d01ac9c87a6d3dbdf56d16d5b7 |
| BCW-000702 | 1.000 | LITG01000066.1 | Lactococcus lactis subsp. cremoris strain NCDO763 | 0da443d01ac9c87a6d3dbdf56d16d5b7 |
| BCW-000703 | 1.000 | LITG01000066.1 | Lactococcus lactis subsp. cremoris strain NCDO763 | 0da443d01ac9c87a6d3dbdf56d16d5b7 |
| BCW-000704 | 1.000 | LITG01000066.1 | Lactococcus lactis subsp. cremoris strain NCDO763 | 0da443d01ac9c87a6d3dbdf56d16d5b7 |
| BCW-000705 | 1.000 | LITG01000066.1 | Lactococcus lactis subsp. cremoris strain NCDO763 | 0da443d01ac9c87a6d3dbdf56d16d5b7 |
| BCW-000706 | 1.000 | LITG01000066.1 | Lactococcus lactis subsp. cremoris strain NCDO763 | 0da443d01ac9c87a6d3dbdf56d16d5b7 |
| BCW-000707 | 1.000 | LITG01000066.1 | Lactococcus lactis subsp. cremoris strain NCDO763 | 0da443d01ac9c87a6d3dbdf56d16d5b7 |
| BCW-000708 | 1.000 | LITG01000066.1 | Lactococcus lactis subsp. cremoris strain NCDO763 | 0da443d01ac9c87a6d3dbdf56d16d5b7 |
| BCW-000709 | 1.000 | LITG01000066.1 | Lactococcus lactis subsp. cremoris strain NCDO763 | 0da443d01ac9c87a6d3dbdf56d16d5b7 |
| BCW-000710 | 1.000 | LITG01000066.1 | Lactococcus lactis subsp. cremoris strain NCDO763 | 0da443d01ac9c87a6d3dbdf56d16d5b7 |
| BCW-000711 | 1.000 | LITG01000066.1 | Lactococcus lactis subsp. cremoris strain NCDO763 | 0da443d01ac9c87a6d3dbdf56d16d5b7 |
| BCW-000712 | 1.000 | LITG01000066.1 | Lactococcus lactis subsp. cremoris strain NCDO763 | 0da443d01ac9c87a6d3dbdf56d16d5b7 |
| BCW-000714 | 1.000 | LITG01000066.1 | Lactococcus lactis subsp. cremoris strain NCDO763 | 0da443d01ac9c87a6d3dbdf56d16d5b7 |
| BCW-000716 | 1.000 | LITG01000066.1 | Lactococcus lactis subsp. cremoris strain NCDO763 | 0da443d01ac9c87a6d3dbdf56d16d5b7 |
| BCW-000717 | 1.000 | LITG01000066.1 | Lactococcus lactis subsp. cremoris strain NCDO763 | 0da443d01ac9c87a6d3dbdf56d16d5b7 |
| BCW-000718 | 1.000 | LITG01000066.1 | Lactococcus lactis subsp. cremoris strain NCDO763 | 0da443d01ac9c87a6d3dbdf56d16d5b7 |
| BCW-000719 | 1.000 | LITG01000066.1 | Lactococcus lactis subsp. cremoris strain NCDO763 | 0da443d01ac9c87a6d3dbdf56d16d5b7 |
| BCW-000722 | 0.988 | LITG01000066.1 | Lactococcus lactis subsp. cremoris strain NCDO763 | 0da443d01ac9c87a6d3dbdf56d16d5b7 |
| BCW-000724 | 0.971 | LITG01000066.1 | Lactococcus lactis subsp. cremoris strain NCDO763 | 0da443d01ac9c87a6d3dbdf56d16d5b7 |
| BCW-000726 | 0.949 | LKPE01000001.1 | Lactococcus lactis subsp. lactis bv. diacetylactis strain CRL264 | 52df96765200788ed5ffc1d71d8d04a9 |
| BCW-000728 | 0.978 | CP002365.1 | Lactococcus lactis subsp. lactis CV56 | 3f5dbd14b0dba1979aff87a4864acac8 |
| BCW-000729 | 0.960 | CP002365.1 | Lactococcus lactis subsp. lactis CV56 | 3f5dbd14b0dba1979aff87a4864acac8 |
| BCW-000730 | 0.857 | AM406671.1 | Lactococcus lactis subsp. cremoris MG1363 | d859bb5f7c0a26e636d9df3629db0dfc |
| BCW-000732 | 0.946 | AM406671.1 | Lactococcus lactis subsp. cremoris MG1363 | d859bb5f7c0a26e636d9df3629db0dfc |
| BCW-000733 | 0.959 | AM406671.1 | Lactococcus lactis subsp. cremoris MG1363 | d859bb5f7c0a26e636d9df3629db0dfc |
| BCW-000735 | 0.932 | LITG01000066.1 | Lactococcus lactis subsp. cremoris strain NCDO763 | 0da443d01ac9c87a6d3dbdf56d16d5b7 |
| BCW-000737 | 0.993 | LKLY01000093.1 | Lactococcus lactis subsp. lactis strain UC317 | aac51cbe7ce41bd235191b20f05c6515 |
| BCW-200051 | 0.573 | LKLU01000066.1 | Lactococcus lactis subsp. lactis strain M20 | 1c93797fcd24b6f9a51129fb829c70e4 |
| BCW-200077 | 0.531 | LKLF01000031.1 | Lactococcus lactis subsp. lactis strain K337 | 3b0d020863b597dbd421c0e6e6db1077 |
| BCW-200121 | 0.577 | LKLF01000031.1 | Lactococcus lactis subsp. lactis strain K337 | 3b0d020863b597dbd421c0e6e6db1077 |
| BCW-200128 | 0.583 | JRFX01000001.1 | Lactococcus lactis strain Bpl1 | 8bb572e8e35407522ac8e8f9ce288b04 |
| BCW-200138 | 0.583 | JRFX01000001.1 | Lactococcus lactis strain Bpl1 | 8bb572e8e35407522ac8e8f9ce288b04 |
| BCW-200150 | 0.833 | LKLL01000017.1 | Lactococcus lactis subsp. lactis strain KF196 | 5f2d2feca5aac8d7ac41177a9943dc16 |
| BCW-200158 | 0.435 | CP004884.1 | Lactococcus lactis subsp. cremoris KW2 | 1071abb9445adbab69d634a2a520d4a3 |
| BCW-200159 | 0.435 | CP004884.1 | Lactococcus lactis subsp. cremoris KW2 | 1071abb9445adbab69d634a2a520d4a3 |
| BCW-200160 | 0.435 | CP004884.1 | Lactococcus lactis subsp. cremoris KW2 | 1071abb9445adbab69d634a2a520d4a3 |
| BCW-200163 | 0.435 | CP004884.1 | Lactococcus lactis subsp. cremoris KW2 | 1071abb9445adbab69d634a2a520d4a3 |
| BCW-200174 | 0.435 | CP004884.1 | Lactococcus lactis subsp. cremoris KW2 | 1071abb9445adbab69d634a2a520d4a3 |
| BCW-200175 | 0.583 | JRFX01000001.1 | Lactococcus lactis strain Bpl1 | 8bb572e8e35407522ac8e8f9ce288b04 |
| BCW-200180 | 0.524 | LKLF01000031.1 | Lactococcus lactis subsp. lactis strain K337 | 3b0d020863b597dbd421c0e6e6db1077 |
| BCW-200188 | 0.586 | CP009054.1 | Lactococcus lactis subsp. lactis NCDO 2118 | 9b7fd1466feda4bb388b77d7832fcf9d |
| BCW-200192 | 0.586 | CP009054.1 | Lactococcus lactis subsp. lactis NCDO 2118 | 9b7fd1466feda4bb388b77d7832fcf9d |
| BCW-200196 | 0.482 | LKLU01000066.1 | Lactococcus lactis subsp. lactis strain M20 | 1c93797fcd24b6f9a51129fb829c70e4 |
| BCW-200198 | 0.510 | LKLF01000031.1 | Lactococcus lactis subsp. lactis strain K337 | 3b0d020863b597dbd421c0e6e6db1077 |
| BCW-200229 | 0.586 | CP009054.1 | Lactococcus lactis subsp. lactis NCDO 2118 | 9b7fd1466feda4bb388b77d7832fcf9d |
| BCW-200232 | 0.586 | CP009054.1 | Lactococcus lactis subsp. lactis NCDO 2118 | 9b7fd1466feda4bb388b77d7832fcf9d |
| BCW-200238 | 0.512 | LKLF01000031.1 | Lactococcus lactis subsp. lactis strain K337 | 3b0d020863b597dbd421c0e6e6db1077 |
| BCW-200241 | 0.521 | LKLF01000031.1 | Lactococcus lactis subsp. lactis strain K337 | 3b0d020863b597dbd421c0e6e6db1077 |
| BCW-201453 | 0.590 | LKLU01000066.1 | Lactococcus lactis subsp. lactis strain M20 | 1c93797fcd24b6f9a51129fb829c70e4 |
| BCW-201861 | 0.992 | AE005176.1 | Lactococcus lactis subsp. lactis Il1403 | 6490bef77c207ccdd87b638e1f7076d0 |

Draft genome assemblies for each *Lactococcus* isolate were used as inputs for Sourmash 3.1.0 [4] to make MinHash signatures with a k-mer size of 51. Genome signatures were queried against a GenBank microbial genome reference database with signatures of equivalent k-mer size (See Methods). Similarity indicates the calculated Jaccard Index between query and reference genome signatures. Similarity values range over a scale of zero to one, where a similarity of 1 demonstrates intersection of all k-mers comprising the union of k-mers between query and reference.

**Table S3. BNF-associated genes identified by Pan-GWAS**

| **Gene** | **Alt. Name** | **Annotation** | **N pos present in** | **N neg present in** | **N pos not present in** | **N neg not present in** | **Odds ratio** | **BH *p*-value** |
| --- | --- | --- | --- | --- | --- | --- | --- | --- |
| group_2085 |  | hypothetical protein | 23 | 3 | 0 | 67 | Inf | 2.04E-15 |
| pspA |  | Phosphoserine phosphatase 1 | 23 | 3 | 0 | 67 | Inf | 2.04E-15 |
| ybiR |  | Inner membrane protein YbiR | 23 | 3 | 0 | 67 | Inf | 2.04E-15 |
| ddrA |  | Single-stranded DNA-binding protein DdrA | 0 | 64 | 23 | 6 | 0 | 2.80E-13 |
| group_1931 |  | hypothetical protein | 0 | 60 | 23 | 10 | 0 | 3.83E-11 |
| xylT_2 |  | D-xylose transporter | 20 | 3 | 3 | 67 | 148.888889 | 3.83E-11 |
| btuD_9 |  | Vitamin B12 import ATP-binding protein BtuD | 17 | 0 | 6 | 70 | Inf | 5.15E-11 |
| group_8190 |  | hypothetical protein | 17 | 0 | 6 | 70 | Inf | 5.15E-11 |
| group_1077 | xerD_1 | Tyrosine recombinase XerD | 17 | 0 | 6 | 70 | Inf | 5.15E-11 |
| group_1791 |  | hypothetical protein | 17 | 0 | 6 | 70 | Inf | 5.15E-11 |
| group_1810 |  | hypothetical protein | 0 | 57 | 23 | 13 | 0 | 2.98E-10 |
| group_2310 |  | hypothetical protein | 0 | 57 | 23 | 13 | 0 | 2.98E-10 |
| group_298 |  | hypothetical protein | 0 | 57 | 23 | 13 | 0 | 2.98E-10 |
| besA |  | Ferri-bacillibactin esterase BesA | 16 | 0 | 7 | 70 | Inf | 2.98E-10 |
| feuB |  | Iron-uptake system permease protein FeuB | 16 | 0 | 7 | 70 | Inf | 2.98E-10 |
| yusV |  | putative siderophore transport system ATP-binding protein YusV | 16 | 0 | 7 | 70 | Inf | 2.98E-10 |
| group_3929 |  | hypothetical protein | 16 | 0 | 7 | 70 | Inf | 2.98E-10 |
| feuA |  | Iron-uptake system-binding protein | 16 | 0 | 7 | 70 | Inf | 2.98E-10 |
| feuC_1 |  | Iron-uptake system permease protein FeuC | 16 | 0 | 7 | 70 | Inf | 2.98E-10 |
| group_2948 |  | hypothetical protein | 17 | 1 | 6 | 69 | 195.5 | 3.73E-10 |
| bcsA |  | Cellulose synthase catalytic subunit [UDP-forming] | 17 | 1 | 6 | 69 | 195.5 | 3.73E-10 |
| group_5428 |  | hypothetical protein | 17 | 1 | 6 | 69 | 195.5 | 3.73E-10 |
| group_7758 |  | hypothetical protein | 17 | 1 | 6 | 69 | 195.5 | 3.73E-10 |
| xerD_1 |  | Tyrosine recombinase XerD | 0 | 56 | 23 | 14 | 0 | 6.00E-10 |
| group_3091 |  | hypothetical protein | 0 | 55 | 23 | 15 | 0 | 1.14E-09 |
| group_6113 |  | hypothetical protein | 18 | 3 | 5 | 67 | 80.4 | 1.14E-09 |
| ybjI_2 |  | 5-amino-6-(5-phospho-D-ribitylamino)uracil phosphatase YbjI | 18 | 3 | 5 | 67 | 80.4 | 1.14E-09 |
| group_6282 | lytR_2 | Transcriptional regulator LytR | 18 | 3 | 5 | 67 | 80.4 | 1.14E-09 |
| group_1352 |  | hypothetical protein | 18 | 3 | 5 | 67 | 80.4 | 1.14E-09 |
| group_6283 |  | hypothetical protein | 18 | 3 | 5 | 67 | 80.4 | 1.14E-09 |
| group_2666 |  | hypothetical protein | 18 | 3 | 5 | 67 | 80.4 | 1.14E-09 |
| group_6284 |  | hypothetical protein | 18 | 3 | 5 | 67 | 80.4 | 1.14E-09 |
| group_2667 |  | hypothetical protein | 18 | 3 | 5 | 67 | 80.4 | 1.14E-09 |
| group_6285 | yagU | Inner membrane protein YagU | 18 | 3 | 5 | 67 | 80.4 | 1.14E-09 |
| group_4863 |  | hypothetical protein | 18 | 3 | 5 | 67 | 80.4 | 1.14E-09 |
| ybjI_1 |  | 5-amino-6-(5-phospho-D-ribitylamino)uracil phosphatase YbjI | 18 | 3 | 5 | 67 | 80.4 | 1.14E-09 |
| tauB |  | Taurine import ATP-binding protein TauB | 18 | 3 | 5 | 67 | 80.4 | 1.14E-09 |
| group_6294 |  | hypothetical protein | 18 | 3 | 5 | 67 | 80.4 | 1.14E-09 |
| maa_1 |  | Maltose O-acetyltransferase | 18 | 3 | 5 | 67 | 80.4 | 1.14E-09 |
| group_5543 |  | hypothetical protein | 15 | 0 | 8 | 70 | Inf | 1.35E-09 |
| group_1786 |  | hypothetical protein | 15 | 0 | 8 | 70 | Inf | 1.35E-09 |
| group_3301 |  | hypothetical protein | 6 | 68 | 17 | 2 | 0.01038062 | 1.80E-09 |
| rbsK/rbiA |  | Bifunctional ribokinase/ribose-5-phosphate isomerase A | 16 | 1 | 7 | 69 | 157.714286 | 1.85E-09 |
| group_7755 | iolU_1 | scyllo-inositol 2-dehydrogenase (NADP(+)) IolU | 16 | 1 | 7 | 69 | 157.714286 | 1.85E-09 |
| group_7756 | ybbH_1 | putative HTH-type transcriptional regulator YbbH | 16 | 1 | 7 | 69 | 157.714286 | 1.85E-09 |
| hsrA_1 |  | putative transport protein HsrA | 16 | 1 | 7 | 69 | 157.714286 | 1.85E-09 |
| group_1660 |  | hypothetical protein | 0 | 54 | 23 | 16 | 0 | 1.85E-09 |
| group_5122 |  | IS21 family transposase IS712 | 0 | 54 | 23 | 16 | 0 | 1.85E-09 |
| licC |  | hypothetical protein | 0 | 53 | 23 | 17 | 0 | 4.18E-09 |
| nhaK |  | hypothetical protein | 23 | 17 | 0 | 53 | Inf | 4.18E-09 |
| group_1288 | pgdA | Peptidoglycan-N-acetylglucosamine deacetylase | 17 | 3 | 6 | 67 | 63.2777778 | 8.66E-09 |
| group_6190 |  | hypothetical protein | 17 | 3 | 6 | 67 | 63.2777778 | 8.66E-09 |
| group_729 | xylA_1 | Reducing end xylose-releasing exo-oligoxylanase | 17 | 3 | 6 | 67 | 63.2777778 | 8.66E-09 |
| group_1379 | dauA | C4-dicarboxylic acid transporter DauA | 0 | 52 | 23 | 18 | 0 | 8.66E-09 |
| group_955 |  | hypothetical protein | 0 | 52 | 23 | 18 | 0 | 8.66E-09 |
| group_2959 |  | hypothetical protein | 14 | 0 | 9 | 70 | Inf | 8.67E-09 |
| group_2687 |  | hypothetical protein | 16 | 2 | 7 | 68 | 77.7142857 | 1.27E-08 |
| group_7770 |  | hypothetical protein | 15 | 1 | 8 | 69 | 129.375 | 1.38E-08 |
| group_407 |  | hypothetical protein | 1 | 55 | 22 | 15 | 0.01239669 | 2.12E-08 |
| group_630 |  | hypothetical protein | 0 | 50 | 23 | 20 | 0 | 3.48E-08 |
| group_1053 |  | hypothetical protein | 0 | 50 | 23 | 20 | 0 | 3.48E-08 |
| group_4010 |  | hypothetical protein | 0 | 50 | 23 | 20 | 0 | 3.48E-08 |
| group_5114 |  | hypothetical protein | 0 | 50 | 23 | 20 | 0 | 3.48E-08 |
| group_5681 |  | hypothetical protein | 0 | 50 | 23 | 20 | 0 | 3.48E-08 |
| group_5682 |  | hypothetical protein | 0 | 50 | 23 | 20 | 0 | 3.48E-08 |
| licC_3 |  | Lichenan permease IIC component | 5 | 64 | 18 | 6 | 0.02604167 | 5.54E-08 |
| group_8447 |  | hypothetical protein | 13 | 0 | 10 | 70 | Inf | 5.79E-08 |
| group_884 | metI | Cystathionine gamma-synthase/O-acetylhomoserine (thiol)-lyase | 0 | 49 | 23 | 21 | 0 | 6.50E-08 |
| group_2066 | xerD_1 | Tyrosine recombinase XerD | 0 | 49 | 23 | 21 | 0 | 6.50E-08 |
| group_4070 |  | hypothetical protein | 0 | 49 | 23 | 21 | 0 | 6.50E-08 |
| group_4357 |  | hypothetical protein | 0 | 49 | 23 | 21 | 0 | 6.50E-08 |
| yxeP_1 |  | putative hydrolase YxeP | 0 | 49 | 23 | 21 | 0 | 6.50E-08 |
| group_5879 |  | hypothetical protein | 0 | 49 | 23 | 21 | 0 | 6.50E-08 |
| araA |  | L-arabinose isomerase | 15 | 2 | 8 | 68 | 63.75 | 7.87E-08 |
| araB |  | Ribulokinase | 15 | 2 | 8 | 68 | 63.75 | 7.87E-08 |
| araD |  | L-ribulose-5-phosphate 4-epimerase AraD | 15 | 2 | 8 | 68 | 63.75 | 7.87E-08 |
| xylT_1 |  | D-xylose transporter | 15 | 2 | 8 | 68 | 63.75 | 7.87E-08 |
| araR_1 |  | Arabinose metabolism transcriptional repressor | 15 | 2 | 8 | 68 | 63.75 | 7.87E-08 |
| group_6073 |  | hypothetical protein | 17 | 5 | 6 | 65 | 36.8333333 | 1.09E-07 |
| group_6074 | degA_2 | HTH-type transcriptional regulator DegA | 17 | 5 | 6 | 65 | 36.8333333 | 1.09E-07 |
| gmuE_1 |  | Putative fructokinase | 17 | 5 | 6 | 65 | 36.8333333 | 1.09E-07 |
| scrB |  | Sucrose-6-phosphate hydrolase | 17 | 5 | 6 | 65 | 36.8333333 | 1.09E-07 |
| group_2985 |  | hypothetical protein | 23 | 22 | 0 | 48 | Inf | 1.09E-07 |
| lacR |  | Lactose phosphotransferase system repressor | 0 | 48 | 23 | 22 | 0 | 1.09E-07 |
| lacC_1 |  | Tagatose-6-phosphate kinase | 0 | 48 | 23 | 22 | 0 | 1.09E-07 |
| group_3486 | lacA_1 | Galactose-6-phosphate isomerase subunit LacA | 0 | 48 | 23 | 22 | 0 | 1.09E-07 |
| lacB |  | Galactose-6-phosphate isomerase subunit LacB | 0 | 48 | 23 | 22 | 0 | 1.09E-07 |
| xpkA |  | Xylulose-5-phosphate phosphoketolase | 23 | 22 | 0 | 48 | Inf | 1.09E-07 |
| group_2984 |  | hypothetical protein | 23 | 22 | 0 | 48 | Inf | 1.09E-07 |
| xerD_2 |  | Tyrosine recombinase XerD | 16 | 4 | 7 | 66 | 37.7142857 | 1.35E-07 |
| group_2270 |  | hypothetical protein | 0 | 47 | 23 | 23 | 0 | 1.35E-07 |
| group_3569 |  | hypothetical protein | 0 | 47 | 23 | 23 | 0 | 1.35E-07 |
| lacD |  | Tagatose 1,6-diphosphate aldolase | 0 | 47 | 23 | 23 | 0 | 1.35E-07 |
| group_5104 |  | hypothetical protein | 0 | 47 | 23 | 23 | 0 | 1.35E-07 |
| group_731 | bglH_1 | Aryl-phospho-beta-D-glucosidase BglH | 0 | 47 | 23 | 23 | 0 | 1.35E-07 |
| arcC1_3 |  | Carbamate kinase 1 | 23 | 23 | 0 | 47 | Inf | 1.35E-07 |
| group_2272 |  | hypothetical protein | 0 | 47 | 23 | 23 | 0 | 1.35E-07 |
| group_3573 |  | hypothetical protein | 0 | 47 | 23 | 23 | 0 | 1.35E-07 |
| group_4930 |  | hypothetical protein | 0 | 47 | 23 | 23 | 0 | 1.35E-07 |
| clpP_2 |  | ATP-dependent Clp protease proteolytic subunit | 0 | 47 | 23 | 23 | 0 | 1.35E-07 |
| group_1219 |  | hypothetical protein | 23 | 23 | 0 | 47 | Inf | 1.35E-07 |
| group_4133 |  | hypothetical protein | 23 | 23 | 0 | 47 | Inf | 1.35E-07 |
| comEC_1 |  | ComE operon protein 3 | 0 | 47 | 23 | 23 | 0 | 1.35E-07 |
| patB |  | Cystathionine beta-lyase PatB | 0 | 47 | 23 | 23 | 0 | 1.35E-07 |
| group_3579 |  | hypothetical protein | 0 | 47 | 23 | 23 | 0 | 1.35E-07 |
| group_4936 |  | hypothetical protein | 0 | 47 | 23 | 23 | 0 | 1.35E-07 |
| degA_1 |  | HTH-type transcriptional regulator DegA | 0 | 47 | 23 | 23 | 0 | 1.35E-07 |
| group_2375 |  | putative oxidoreductase | 23 | 23 | 0 | 47 | Inf | 1.35E-07 |
| aguA |  | Putative agmatine deiminase | 23 | 23 | 0 | 47 | Inf | 1.35E-07 |
| group_1101 | glxR | 2-hydroxy-3-oxopropionate reductase | 0 | 47 | 23 | 23 | 0 | 1.35E-07 |
| group_289 |  | hypothetical protein | 0 | 47 | 23 | 23 | 0 | 1.35E-07 |
| group_3612 |  | hypothetical protein | 0 | 47 | 23 | 23 | 0 | 1.35E-07 |
| group_4996 |  | hypothetical protein | 0 | 47 | 23 | 23 | 0 | 1.35E-07 |
| group_6331 |  | hypothetical protein | 0 | 47 | 23 | 23 | 0 | 1.35E-07 |
| group_2401 |  | hypothetical protein | 23 | 23 | 0 | 47 | Inf | 1.35E-07 |
| group_4611 |  | hypothetical protein | 23 | 23 | 0 | 47 | Inf | 1.35E-07 |
| group_1308 |  | hypothetical protein | 0 | 47 | 23 | 23 | 0 | 1.35E-07 |
| group_2977 |  | hypothetical protein | 0 | 47 | 23 | 23 | 0 | 1.35E-07 |
| group_3801 |  | hypothetical protein | 0 | 47 | 23 | 23 | 0 | 1.35E-07 |
| group_4997 |  | hypothetical protein | 0 | 47 | 23 | 23 | 0 | 1.35E-07 |
| group_6413 |  | hypothetical protein | 0 | 47 | 23 | 23 | 0 | 1.35E-07 |
| araR |  | Arabinose metabolism transcriptional repressor | 23 | 23 | 0 | 47 | Inf | 1.35E-07 |
| mngB |  | Mannosylglycerate hydrolase | 23 | 23 | 0 | 47 | Inf | 1.35E-07 |
| mntH_2 |  | Divalent metal cation transporter MntH | 0 | 47 | 23 | 23 | 0 | 1.35E-07 |
| rpe_1 |  | Ribulose-phosphate 3-epimerase | 0 | 47 | 23 | 23 | 0 | 1.35E-07 |
| group_3973 |  | hypothetical protein | 0 | 47 | 23 | 23 | 0 | 1.35E-07 |
| group_5009 |  | hypothetical protein | 0 | 47 | 23 | 23 | 0 | 1.35E-07 |
| group_6485 | adhR_2 | HTH-type transcriptional regulator AdhR | 0 | 47 | 23 | 23 | 0 | 1.35E-07 |
| group_2630 |  | hypothetical protein | 23 | 23 | 0 | 47 | Inf | 1.35E-07 |
| group_4613 |  | hypothetical protein | 23 | 23 | 0 | 47 | Inf | 1.35E-07 |
| csbB_1 |  | Putative glycosyltransferase CsbB | 0 | 47 | 23 | 23 | 0 | 1.35E-07 |
| rpiB |  | Ribose-5-phosphate isomerase B | 0 | 47 | 23 | 23 | 0 | 1.35E-07 |
| group_430 |  | hypothetical protein | 0 | 47 | 23 | 23 | 0 | 1.35E-07 |
| tarF |  | Teichoic acid poly(glycerol phosphate) polymerase | 0 | 47 | 23 | 23 | 0 | 1.35E-07 |
| group_6487 |  | hypothetical protein | 0 | 47 | 23 | 23 | 0 | 1.35E-07 |
| group_3337 | yteP | putative multiple-sugar transport system permease YteP | 23 | 23 | 0 | 47 | Inf | 1.35E-07 |
| group_5437 |  | hypothetical protein | 23 | 23 | 0 | 47 | Inf | 1.35E-07 |
| rhaR_2 |  | HTH-type transcriptional activator RhaR | 0 | 47 | 23 | 23 | 0 | 1.35E-07 |
| group_3550 |  | hypothetical protein | 0 | 47 | 23 | 23 | 0 | 1.35E-07 |
| lacF_1 |  | PTS system lactose-specific EIIA component | 0 | 47 | 23 | 23 | 0 | 1.35E-07 |
| group_5103 |  | hypothetical protein | 0 | 47 | 23 | 23 | 0 | 1.35E-07 |
| group_6489 |  | hypothetical protein | 0 | 47 | 23 | 23 | 0 | 1.35E-07 |
| group_3339 | xynB | Beta-xylosidase | 23 | 23 | 0 | 47 | Inf | 1.35E-07 |
| araQ |  | L-arabinose transport system permease protein AraQ | 23 | 23 | 0 | 47 | Inf | 1.35E-07 |
| group_1793 |  | hypothetical protein | 12 | 0 | 11 | 70 | Inf | 1.89E-07 |
| group_3912 |  | hypothetical protein | 12 | 0 | 11 | 70 | Inf | 1.89E-07 |
| group_5483 |  | hypothetical protein | 12 | 0 | 11 | 70 | Inf | 1.89E-07 |
| group_5487 |  | hypothetical protein | 12 | 0 | 11 | 70 | Inf | 1.89E-07 |
| group_5497 |  | hypothetical protein | 12 | 0 | 11 | 70 | Inf | 1.89E-07 |
| group_5560 |  | hypothetical protein | 12 | 0 | 11 | 70 | Inf | 1.89E-07 |
| group_8253 |  | hypothetical protein | 12 | 0 | 11 | 70 | Inf | 1.89E-07 |
| crr_1 |  | hypothetical protein | 1 | 51 | 22 | 19 | 0.01693405 | 2.00E-07 |
| group_1839 | licC_2 | Lichenan permease IIC component | 18 | 8 | 5 | 62 | 27.9 | 2.40E-07 |
| group_868 |  | hypothetical protein | 13 | 1 | 10 | 69 | 89.7 | 2.97E-07 |
| group_1351 |  | hypothetical protein | 13 | 1 | 10 | 69 | 89.7 | 2.97E-07 |
| group_894 | cspL | Cold shock protein 2 | 13 | 1 | 10 | 69 | 89.7 | 2.97E-07 |
| group_5436 | gtf1_1 | Glycosyltransferase Gtf1 | 13 | 1 | 10 | 69 | 89.7 | 2.97E-07 |
| group_696 |  | hypothetical protein | 13 | 1 | 10 | 69 | 89.7 | 2.97E-07 |
| group_7798 |  | hypothetical protein | 13 | 1 | 10 | 69 | 89.7 | 2.97E-07 |
| group_7799 | lacF_2 | Lactose transport system permease protein LacF | 13 | 1 | 10 | 69 | 89.7 | 2.97E-07 |
| group_7961 | btuD_6 | Vitamin B12 import ATP-binding protein BtuD | 13 | 1 | 10 | 69 | 89.7 | 2.97E-07 |
| group_8186 |  | hypothetical protein | 13 | 1 | 10 | 69 | 89.7 | 2.97E-07 |
| xynB |  | Beta-xylosidase | 0 | 46 | 23 | 24 | 0 | 3.14E-07 |
| group_5085 |  | hypothetical protein | 0 | 46 | 23 | 24 | 0 | 3.14E-07 |
| group_2271 |  | hypothetical protein | 0 | 46 | 23 | 24 | 0 | 3.14E-07 |
| pinR |  | Serine recombinase PinR | 0 | 46 | 23 | 24 | 0 | 3.14E-07 |
| group_1295 |  | hypothetical protein | 0 | 46 | 23 | 24 | 0 | 3.14E-07 |
| lacE |  | PTS system lactose-specific EIICB component | 0 | 46 | 23 | 24 | 0 | 3.14E-07 |
| group_6544 |  | hypothetical protein | 0 | 46 | 23 | 24 | 0 | 3.14E-07 |
| group_1306 |  | hypothetical protein | 0 | 46 | 23 | 24 | 0 | 3.14E-07 |
| group_2975 |  | hypothetical protein | 0 | 46 | 23 | 24 | 0 | 3.14E-07 |
| group_6644 |  | hypothetical protein | 0 | 46 | 23 | 24 | 0 | 3.14E-07 |
| licT_1 |  | Transcription antiterminator LicT | 0 | 46 | 23 | 24 | 0 | 3.14E-07 |
| group_2976 |  | hypothetical protein | 0 | 46 | 23 | 24 | 0 | 3.14E-07 |
| group_6691 |  | hypothetical protein | 0 | 46 | 23 | 24 | 0 | 3.14E-07 |
| group_2103 |  | Serine-pyruvate aminotransferase | 0 | 46 | 23 | 24 | 0 | 3.14E-07 |
| group_3566 |  | hypothetical protein | 0 | 46 | 23 | 24 | 0 | 3.14E-07 |
| emrB_3 |  | Multidrug export protein EmrB | 0 | 46 | 23 | 24 | 0 | 3.14E-07 |
| licC_1 |  | Lichenan permease IIC component | 0 | 46 | 23 | 24 | 0 | 3.14E-07 |
| xerD_4 |  | Tyrosine recombinase XerD | 0 | 46 | 23 | 24 | 0 | 3.14E-07 |
| oppC_1 |  | Oligopeptide transport system permease protein OppC | 0 | 46 | 23 | 24 | 0 | 3.14E-07 |
| garK_1 |  | Glycerate 2-kinase | 0 | 46 | 23 | 24 | 0 | 3.14E-07 |
| group_5034 |  | hypothetical protein | 0 | 46 | 23 | 24 | 0 | 3.14E-07 |
| group_2305 |  | IS3 family transposase IS1076 | 0 | 45 | 23 | 25 | 0 | 4.35E-07 |
| group_2720 |  | hypothetical protein | 0 | 45 | 23 | 25 | 0 | 4.35E-07 |
| pcp |  | Pyrrolidone-carboxylate peptidase | 0 | 45 | 23 | 25 | 0 | 4.35E-07 |
| lacX_2 |  | Protein LacX, plasmid | 0 | 45 | 23 | 25 | 0 | 4.35E-07 |
| lacG |  | 6-phospho-beta-galactosidase | 0 | 45 | 23 | 25 | 0 | 4.35E-07 |
| group_917 | azoR2 | FMN-dependent NADH-azoreductase 2 | 0 | 45 | 23 | 25 | 0 | 4.35E-07 |
| comGA |  | ComG operon protein 1 | 0 | 45 | 23 | 25 | 0 | 4.35E-07 |
| group_5222 |  | hypothetical protein | 17 | 7 | 6 | 63 | 25.5 | 5.31E-07 |
| group_624 |  | hypothetical protein | 18 | 9 | 5 | 61 | 24.4 | 5.35E-07 |
| group_1594 |  | hypothetical protein | 0 | 44 | 23 | 26 | 0 | 7.12E-07 |
| dld |  | Quinone-dependent D-lactate dehydrogenase | 0 | 44 | 23 | 26 | 0 | 7.12E-07 |
| group_2442 |  | hypothetical protein | 0 | 44 | 23 | 26 | 0 | 7.12E-07 |
| group_3159 |  | hypothetical protein | 0 | 44 | 23 | 26 | 0 | 7.12E-07 |
| group_377 |  | hypothetical protein | 0 | 44 | 23 | 26 | 0 | 7.12E-07 |
| pepO_1 |  | Neutral endopeptidase | 0 | 44 | 23 | 26 | 0 | 7.12E-07 |
| group_5251 |  | hypothetical protein | 0 | 44 | 23 | 26 | 0 | 7.12E-07 |
| group_608 |  | hypothetical protein | 0 | 44 | 23 | 26 | 0 | 7.12E-07 |
| group_1346 |  | hypothetical protein | 11 | 0 | 12 | 70 | Inf | 9.13E-07 |
| group_5503 |  | hypothetical protein | 11 | 0 | 12 | 70 | Inf | 9.13E-07 |
| group_173 |  | hypothetical protein | 11 | 0 | 12 | 70 | Inf | 9.13E-07 |
| bltD |  | Spermine/spermidine acetyltransferase | 11 | 0 | 12 | 70 | Inf | 9.13E-07 |
| group_2257 |  | hypothetical protein | 11 | 0 | 12 | 70 | Inf | 9.13E-07 |
| group_5623 | prmC_1 | Release factor glutamine methyltransferase | 11 | 0 | 12 | 70 | Inf | 9.13E-07 |
| group_2262 |  | hypothetical protein | 11 | 0 | 12 | 70 | Inf | 9.13E-07 |
| group_5626 |  | hypothetical protein | 11 | 0 | 12 | 70 | Inf | 9.13E-07 |
| group_2960 |  | hypothetical protein | 11 | 0 | 12 | 70 | Inf | 9.13E-07 |
| group_764 |  | hypothetical protein | 11 | 0 | 12 | 70 | Inf | 9.13E-07 |
| group_2965 |  | hypothetical protein | 11 | 0 | 12 | 70 | Inf | 9.13E-07 |
| group_8270 |  | hypothetical protein | 11 | 0 | 12 | 70 | Inf | 9.13E-07 |
| group_301 |  | hypothetical protein | 11 | 0 | 12 | 70 | Inf | 9.13E-07 |
| group_3932 |  | hypothetical protein | 11 | 0 | 12 | 70 | Inf | 9.13E-07 |

Gene indicates the ID assigned to the group of homologous protein coding sequences determined through annotation and pangenome analysis with Roary [5,6]. Annotations reflect descriptions generated by Prokka 1.12 using default settings. Abbreviations describing summaries of gene presence and absence information include: N, Number; pos, positive; neg, negative. The term positive refers to isolates designated as diazotrophic and negative to those assigned to the control population. Odds ratios and Benjaminni-Hochberg adjusted *p*-values of significance for each identified gene group were generated using Scoary 1.6.16 [7].

**Table S4. Annotation of BNF-associated genes commonly identified by Pan-GWAS and RF modeling**

| **Gene ID** | **Prokka Annotation** | **Interproscan Annotation** | **Pan-GWAS** | **RFC** | **RFR** | **State** |
| --- | --- | --- | --- | --- | --- | --- |
| araA | L-arabinose isomerase | L-arabinose isomerase | **+** | **+** | **+** | **+** |
| araB | Ribulokinase | Xylulose Kinase | **+** | **+** | **-** | **+** |
| araD | L-ribulose-5-phosphate 4-epimerase AraD | L-Ribulose-5-phosphate 4-epimerase SGBE | **+** | **+** | **-** | **+** |
| araR_1 | Arabinose metabolism transcriptional repressor | Arabinose metabolism transcriptional repressor | **+** | **+** | **-** | **+** |
| arcC1_3 | Carbamate kinase 1 | arcC: carbamate kinase | **+** | **+** | **-** | **+** |
| bcsA | Cellulose synthase catalytic subunit [UDP-forming] | Bacterial cellulose synthase subunit A | **+** | **+** | **-** | **+** |
| besA | Ferri-bacillibactin esterase BesA | Siderophore triacetylfusarinine C esterase | **+** | **+** | **-** | **+** |
| bltD | Spermine/spermidine acetyltransferase | Spermine/Spermidine acetyltransferase | **+** | **+** | **-** | **+** |
| btuD_9 | Vitamin B12 import ATP-binding protein BtuD | ABC Transporter Type 1 | **+** | **+** | **+** | **+** |
| comEC_1 | ComE operon protein 3 | DNA recombination protein | **+** | **+** | **-** | **-** |
| crr_1 | hypothetical protein | PTS system N-acetylmuramic acid-specific EIIBC component | **+** | **+** | **-** | **-** |
| ddrA | Single-stranded DNA-binding protein DdrA | Rad52/22 family double-strand break repair protein | **+** | **+** | **+** | **-** |
| feuA | Iron-uptake system-binding protein | Iron siderophore periplasmic ABC transporter | **+** | **+** | **+** | **+** |
| feuB | Iron-uptake system permease protein FeuB | Transmembrane ABC transporter iron/siderophore pemease | **+** | **+** | **-** | **+** |
| feuC_1 | Iron-uptake system permease protein FeuC | Transmembrane ABC transporter iron/siderophore permease | **+** | **+** | **-** | **+** |
| garK_1 | Glycerate 2-kinase | Glycerate Kinase | **+** | **+** | **-** | **-** |
| gmuB | PTS system oligo-beta-mannoside-specific EIIB component | PTS system N,N'-diacetylchitobiose-specific EIIB component | **-** | **+** | **+** | **+** |
| gmuE_1 | Putative fructokinase | Transcriptional repressor MPRA | **+** | **+** | **-** | **+** |
| group_1077 | Tyrosine recombinase XerD | DNA Integrase/Recombinase | **+** | **+** | **-** | **+** |
| group_1219 | hypothetical protein | Extracellular galactose binding protein | **+** | **+** | **-** | **+** |
| group_1288 | Peptidoglycan-N-acetylglucosamine deacetylase | NodB-like chitooligosaccharide deacetylase | **+** | **+** | **-** | **+** |
| group_1352 | hypothetical protein | Calcineurin-like phosphoesterase | **+** | **+** | **-** | **+** |
| group_1660 | hypothetical protein | No matches | **+** | **+** | **-** | **-** |
| group_173 | hypothetical protein | Bacteriophage peptidoglycan hydrolase / lysin | **+** | **-** | **+** | **+** |
| group_1791 | hypothetical protein | No Matches | **+** | **+** | **+** | **+** |
| group_1793 | hypothetical protein | Putative lactococcus lactis phage r1t holin | **+** | **+** | **+** | **+** |
| group_1810 | hypothetical protein | Protein of unknown function DUF722 | **+** | **+** | **-** | **-** |
| group_1931 | hypothetical protein | Intrinsically disordered protein | **+** | **+** | **+** | **-** |
| group_2066 | Tyrosine recombinase XerD | Phage-like Integrase | **+** | **+** | **-** | **-** |
| group_2085 | hypothetical protein | Glyoxalase/Bleomycin resistance protein/Dihydroxybiphenyl dioxygenase | **+** | **+** | **+** | **+** |
| group_2103 | Serine-pyruvate aminotransferase | Serine-pyruvate aminotransferase/2-aminoethylphosphonate-pyruvate transaminase | **+** | **+** | **-** | **-** |
| group_2262 | hypothetical protein | Intrinsically disordered protein | **+** | **+** | **+** | **+** |
| group_2310 | hypothetical protein | Phage Terminase small subunit | **+** | **+** | **-** | **-** |
| group_2401 | hypothetical protein | Amino acid / polyamine permease transporter | **+** | **+** | **-** | **+** |
| group_2588 | 1-deoxy-D-xylulose-5-phosphate synthase | 1-deoxy-D-xylulose-5-phosphate synthase | **-** | **+** | **+** | **+** |
| group_2666 | hypothetical protein | No Matches | **+** | **+** | **-** | **+** |
| group_2667 | hypothetical protein | Domain of unknown function (DUF4395) | **+** | **+** | **-** | **+** |
| group_2948 | hypothetical protein | EAL domain containing cyclic di-GMP phosphodiesterase PDEI-related | **+** | **+** | **-** | **+** |
| group_2949 | hypothetical protein | intrinsically disordered protein | **-** | **+** | **+** | **+** |
| group_2959 | hypothetical protein | MepB-like Protein of Unknown Function | **+** | **+** | **+** | **+** |
| group_2960 | hypothetical protein | No Matches | **+** | **+** | **+** | **+** |
| group_2965 | hypothetical protein | Intrinsically disordered protein | **+** | **-** | **+** | **+** |
| group_298 | hypothetical protein | Winged helix-like DNA-binding domain superfamily | **+** | **+** | **-** | **-** |
| group_301 | hypothetical protein | Winged helix-like DNA-binding domain superfamily | **+** | **+** | **-** | **+** |
| group_3091 | hypothetical protein | No Matches | **+** | **+** | **-** | **-** |
| group_3096 | hypothetical protein | No Matches | **-** | **+** | **+** | **+** |
| group_3301 | hypothetical protein | WxL domain surface cell wall-binding | **+** | **+** | **+** | **+** |
| group_3903 | Sugar transporter SemiSWEET | Membrane-bound protein with PQ-loop repeats | **-** | **+** | **+** | **+** |
| group_3905 | hypothetical protein | Outer surface protein with aldolase and peptidyl-prolyl isomerase domains | **-** | **+** | **+** | **+** |
| group_3907 | hypothetical protein | No matches | **-** | **+** | **+** | **+** |
| group_3912 | hypothetical protein | No matches | **+** | **+** | **-** | **+** |
| group_3929 | hypothetical protein | F420-Dependent-like NADP oxidoreductase | **+** | **+** | **-** | **+** |
| group_3932 | hypothetical protein | Putative DNA-binding domain superfamily | **+** | **+** | **+** | **+** |
| group_4010 | hypothetical protein | Intrinsically disordered protein | **+** | **+** | **-** | **-** |
| group_407 | hypothetical protein | Intrinsically disordered protein | **+** | **+** | **-** | **-** |
| group_4070 | hypothetical protein | No matches | **+** | **+** | **-** | **-** |
| group_4133 | hypothetical protein | ArsR-type transcriptional regulatory protein | **+** | **+** | **-** | **+** |
| group_4611 | hypothetical protein | Periplasmic G3P-like solute binding protein | **+** | **+** | **-** | **+** |
| group_4863 | hypothetical protein | Metl-like transmembrane ABC transporter type I | **+** | **+** | **-** | **+** |
| group_4996 | hypothetical protein | DNA/RNA non-specific endonuclease | **+** | **+** | **-** | **-** |
| group_4997 | hypothetical protein | Intrinsically disordered protein | **+** | **+** | **-** | **-** |
| group_5122 | IS21 family transposase IS712 | AAA+ ATP hydrolase / Chromosomal replication initiator protein DNAA | **+** | **+** | **-** | **-** |
| group_5428 | hypothetical protein | Winged helix/Mga helix-turn-helix DNA binding | **+** | **+** | **+** | **+** |
| group_5436 | Glycosyltransferase Gtf1 | Membrane-bound galactosyl-transferase / GT1 | **+** | **+** | **+** | **+** |
| group_5437 | hypothetical protein | Alpha-1,2-mannosidase, putative / glycosyl hydrolase family 92 | **+** | **+** | **-** | **+** |
| group_5483 | hypothetical protein | No Matches | **+** | **+** | **+** | **+** |
| group_5487 | hypothetical protein | Protein of unknown function DUF3892 | **+** | **+** | **+** | **+** |
| group_5490 | hypothetical protein | Haemolysin XhlA | **-** | **+** | **+** | **+** |
| group_5497 | hypothetical protein | No Matches | **+** | **+** | **+** | **+** |
| group_5503 | hypothetical protein | No Matches | **+** | **+** | **+** | **+** |
| group_5543 | hypothetical protein | No matches | **+** | **+** | **-** | **+** |
| group_5560 | hypothetical protein | YxeA-like superfamily protein of unknown function DUF1093 | **+** | **-** | **+** | **+** |
| group_5623 | Release factor glutamine methyltransferase | Type I DNA methyltransferase | **+** | **+** | **+** | **+** |
| group_5624 | hypothetical protein | Fido-domain containing AMP-protein transferase | **-** | **+** | **+** | **+** |
| group_5625 | Type I restriction enzyme EcoR124II R protein | Type I restriction endonuclease | **-** | **+** | **+** | **+** |
| group_5626 | hypothetical protein | No Matches | **+** | **+** | **+** | **+** |
| group_5879 | hypothetical protein | Protein of unknown function (DUF722) | **+** | **+** | **-** | **-** |
| group_6073 | hypothetical protein | Phosphotransferase system, sucrose-specific IIBC component | **+** | **+** | **-** | **+** |
| group_6074 | HTH-type transcriptional regulator DegA | Lactose operon repressor / transcription factor | **+** | **+** | **-** | **+** |
| group_6113 | hypothetical protein | Intrinsically disordered protein | **+** | **+** | **-** | **+** |
| group_6190 | hypothetical protein | yocH / RlpA - like cell wall-binding o-glycosyl hydrolase | **+** | **+** | **-** | **+** |
| group_624 | hypothetical protein | Bacterial lectin / B-type Collagen-binding surface protein - Cna-like | **+** | **+** | **-** | **+** |
| group_6282 | Transcriptional regulator LytR | Polyisoprenyl-teichoic acid--peptidoglycan teichoic acid transferase TAGU | **+** | **+** | **-** | **+** |
| group_6283 | hypothetical protein | CPBP intramembrane metalloprotease / CAAX prenyl protease 2 | **+** | **+** | **-** | **+** |
| group_6284 | hypothetical protein | TTHA1013/TTHA0281-like protein with speculated involvement in RNA metabolism | **+** | **+** | **-** | **+** |
| group_6285 | Inner membrane protein YagU | Protein of unknown function (DUF1440) | **+** | **+** | **-** | **+** |
| group_6294 | hypothetical protein | ROK (Repressor, ORF, Kinase) DNA-Binding Transcription Factor | **+** | **+** | **+** | **+** |
| group_6487 | hypothetical protein | No matches | **+** | **+** | **-** | **-** |
| group_696 | hypothetical protein | No Matches | **+** | **+** | **+** | **+** |
| group_729 | Reducing end xylose-releasing exo-oligoxylanase | Glycosyl hydrolase family 8 | **+** | **+** | **-** | **+** |
| group_764 | hypothetical protein | No matches | **+** | **+** | **-** | **+** |
| group_7755 | scyllo-inositol 2-dehydrogenase (NADP(+)) IolU | NADP binding Oxidoreductase | **+** | **+** | **-** | **+** |
| group_7758 | hypothetical protein | GGDEF: diguanylate cyclase | **+** | **+** | **-** | **+** |
| group_7770 | hypothetical protein | Prokaryotic membrane lipoprotein lipid attachment | **+** | **+** | **+** | **+** |
| group_7798 | hypothetical protein | Extracellular arabinose binding protein | **+** | **+** | **+** | **+** |
| group_7799 | Lactose transport system permease protein LacF | Binding-protein-dependent Metl-like inner membrane ABC transporter type 1 | **+** | **-** | **+** | **+** |
| group_7841 | Teichoic acid translocation permease protein TagG | Inner membrane ABC -2 type transport permease | **-** | **+** | **+** | **+** |
| group_7846 | hypothetical protein | Protein of unknown function DUF1275 | **-** | **+** | **+** | **+** |
| group_7961 | Vitamin B12 import ATP-binding protein BtuD | ABC Transporter Type 1 | **+** | **+** | **+** | **+** |
| group_8177 | Acetyl esterase | Arylacetamide deacetylase | **-** | **+** | **+** | **+** |
| group_8186 | hypothetical protein | FEMO COFACTOR BIOSYNTHESIS PROTEIN NIFB | **+** | **+** | **+** | **+** |
| group_8190 | hypothetical protein | RGG_Cterm: transcriptional activator, Rgg/GadR/MutR family | **+** | **+** | **+** | **+** |
| group_8253 | hypothetical protein | Major facilitator superfamily (MFS) transporter | **+** | **-** | **+** | **+** |
| group_8270 | hypothetical protein | No Matches | **+** | **+** | **+** | **+** |
| group_8447 | hypothetical protein | DNA/RNA non-specific endonuclease | **+** | **+** | **-** | **+** |
| group_868 | hypothetical protein | No matches | **+** | **+** | **-** | **+** |
| group_8866 | hypothetical protein | Mga helix-turn-helix domain containing DNA binding protein | **-** | **+** | **+** | **+** |
| group_8868 | hypothetical protein | peptidoglycan-bound (lpxtg motif) mucin-binding protein | **-** | **+** | **+** | **+** |
| group_8871 | hypothetical protein | No Matches | **-** | **+** | **+** | **+** |
| group_894 | Cold shock protein 2 | Cold-shock protein, DNA-binding | **+** | **+** | **-** | **+** |
| group_955 | hypothetical protein | Protein of unknown function (DUF1140) | **+** | **+** | **-** | **-** |
| gtf1 | Glycosyltransferase Gtf1 | TIGR02918: accessory Sec system glycosylation protein GtfA / GT1 | **-** | **+** | **+** | **+** |
| hsrA_1 | putative transport protein HsrA | Tetracycline resistance protein | **+** | **+** | **-** | **+** |
| lacB | Galactose-6-phosphate isomerase subunit LacB | lacB: galactose-6-phosphate isomerase, LacB subunit | **+** | **+** | **-** | **-** |
| lacC_1 | Tagatose-6-phosphate kinase | lacC: tagatose-6-phosphate kinase | **+** | **+** | **-** | **-** |
| lacD | Tagatose 1,6-diphosphate aldolase | lacD: tagatose 1,6-diphosphate aldolase | **+** | **+** | **-** | **-** |
| lacF_1 | PTS system lactose-specific EIIA component | EIIA-LAC: PTS system, lactose-specific IIa component | **+** | **+** | **-** | **-** |
| licC | hypothetical protein | lacE: PTS system, lactose/cellobiose family IIC component | **+** | **+** | **-** | **+** |
| maa_1 | Maltose O-acetyltransferase | Sialic acid synthase related acetyl transferase | **+** | **+** | **-** | **+** |
| nhaK | hypothetical protein | Cation/H+ exchanger, CPA1 family | **+** | **+** | **-** | **+** |
| pinR | Serine recombinase PinR | DNA resolvase / recombinase | **+** | **+** | **-** | **-** |
| pspA | Phosphoserine phosphatase 1 | Histidine Phosphatase | **+** | **+** | **+** | **+** |
| rbsA | Ribose import ATP-binding protein RbsA | D-allose import ABC transporter | **-** | **+** | **+** | **+** |
| rpiB | Ribose-5-phosphate isomerase B | rpiB: ribose 5-phosphate isomerase B | **+** | **+** | **-** | **-** |
| scrB | Sucrose-6-phosphate hydrolase | Sucrose-6-phosphate hydrolase / glycosyl hydrolase family 32 | **+** | **+** | **+** | **+** |
| tauB | Taurine import ATP-binding protein TauB | Taurine ABC transporter | **+** | **+** | **-** | **+** |
| xerD_1 | Tyrosine recombinase XerD | DNA Integrase/Recombinase | **+** | **+** | **-** | **-** |
| xerD_2 | Tyrosine recombinase XerD | Integrase | **+** | **-** | **+** | **+** |
| xylT_1 | D-xylose transporter | SP: MFS transporter, sugar porter (SP) family | **+** | **+** | **+** | **+** |
| xylT_2 | D-xylose transporter | SP: MFS transporter, sugar porter (SP) family | **+** | **+** | **+** | **+** |
| ybiR | Inner membrane protein YbiR | Citrate transporter | **+** | **+** | **+** | **+** |
| ybjI_1 | 5-amino-6-(5-phospho-D-ribitylamino)uracil phosphatase YbjI | Haloacid dehalogenase-like hydrolase / Phosphomannomutase and Phosphatase Like | **+** | **+** | **-** | **+** |
| ybjI_2 | 5-amino-6-(5-phospho-D-ribitylamino)uracil phosphatase YbjI | Haloacid dehalogenase-like hydrolase | **+** | **+** | **-** | **+** |
| yusV | putative siderophore transport system ATP-binding protein YusV | Iron Siderophore / Vitamin B12 / Hemin ABC transporter | **+** | **+** | **-** | **+** |
| yxeP_1 | putative hydrolase YxeP | Amidohydrolase / bacterial exopeptidase | **+** | **+** | **-** | **-** |

Gene ID indicates the reference designated for the cluster of homologous genes identified through Prokka 1.12 [5] annotation of individual isolate genomes and grouping with Roary 3.12.0 [6]. Functional descriptions provided by Interproscan were presented as manual inference based on interpretation of domain hits for each protein to multiple reference databases queried in the annotation pipeline. Raw output from Interproscan runs were made available online (see Methods). Pan-GWAS, Random Forests classification (RFC) and Random Forests regression (RFR) models were used to identify each of the genes presented in the table. Determination of importance for the BNF trait by the respective computational approach was indicated using a (+), while lack of detection was indicated with (-). State indicates presence (+) or absence (-) of the gene in diazotrophic *L. lactis* isolate genomes.

## References

1. Conway, J.R.; Lex, A.; Gehlenborg, N. UpSetR: an R package for the visualization of intersecting sets and their properties. *Bioinformatics* **2017**, *33*, 2938-2940, doi:10.1093/bioinformatics/btx364.

2. Li, D.; Liu, C.M.; Luo, R.; Sadakane, K.; Lam, T.W. MEGAHIT: an ultra-fast single-node solution for large and complex metagenomics assembly via succinct de Bruijn graph. *Bioinformatics* **2015**, *31*, 1674-1676, doi:10.1093/bioinformatics/btv033.

3. Gurevich, A.; Saveliev, V.; Vyahhi, N.; Tesler, G. QUAST: quality assessment tool for genome assemblies. *Bioinformatics* **2013**, *29*, 1072-1075, doi:10.1093/bioinformatics/btt086.

4. Brown, C.T.; Irber, L. sourmash: a library for MinHash sketching of DNA. *J. Open Source Software* **2016**, *1*, 27, doi:10.21105/joss.00027.

5. Seemann, T. Prokka: rapid prokaryotic genome annotation. *Bioinformatics* **2014**, *30*, 2068-2069, doi:10.1093/bioinformatics/btu153.

6. Page, A.J.; Cummins, C.A.; Hunt, M.; Wong, V.K.; Reuter, S.; Holden, M.T.; Fookes, M.; Falush, D.; Keane, J.A.; Parkhill, J. Roary: rapid large-scale prokaryote pan genome analysis. *Bioinformatics* **2015**, *31*, 3691-3693, doi:10.1093/bioinformatics/btv421.

7. Brynildsrud, O.; Bohlin, J.; Scheffer, L.; Eldholm, V. Rapid scoring of genes in microbial pan-genome-wide association studies with Scoary. *Genome Biol* **2016**, *17*, 238, doi:10.1186/s13059-016-1108-8.
