# Supplementary figures and images for: Identification of Nitrogen Fixation Genes in Lactococcus Isolated from Maize Using Population Genomics and Machine Learning
Source: Microorganisms. 2020 Dec 20;8(12):2043. doi: 10.3390/microorganisms8122043 (PMC7768417; doi:10.3390/microorganisms8122043)

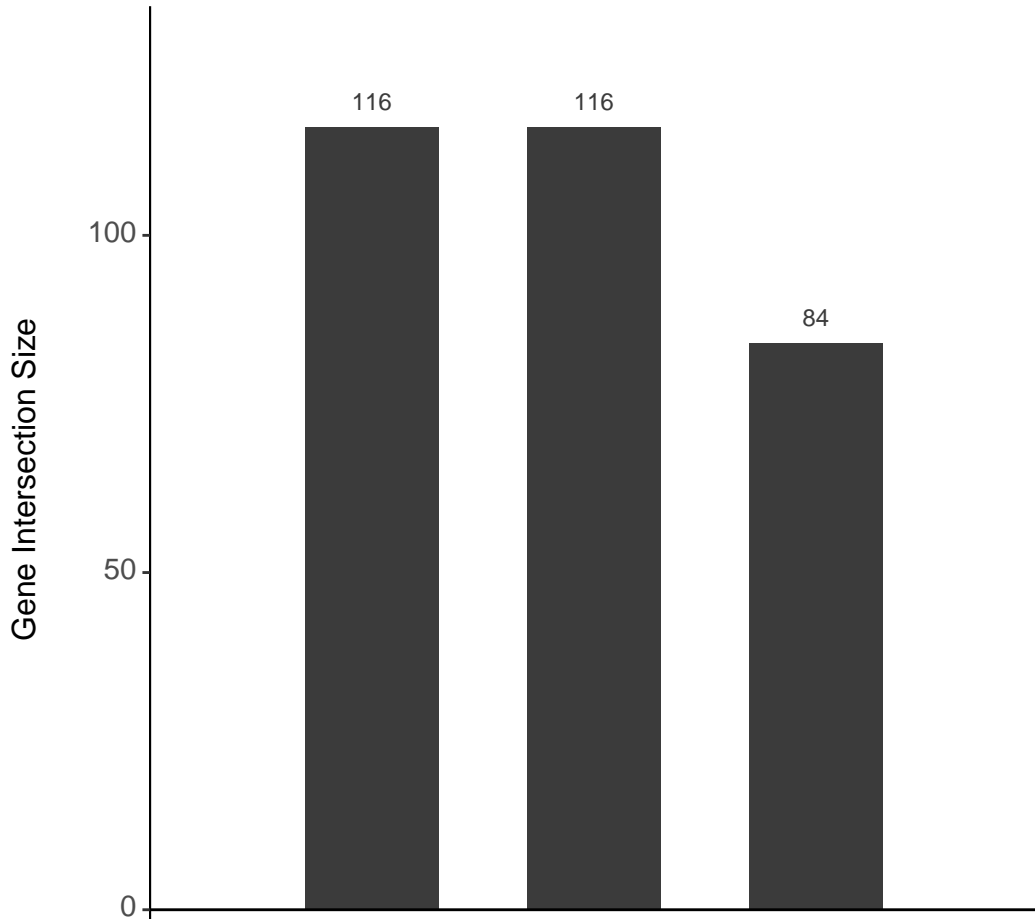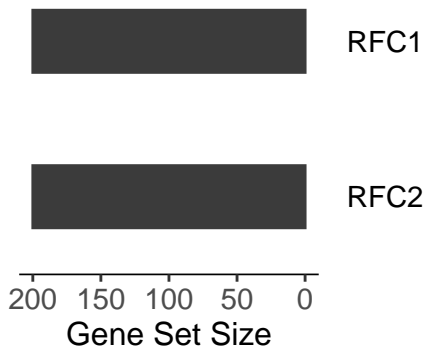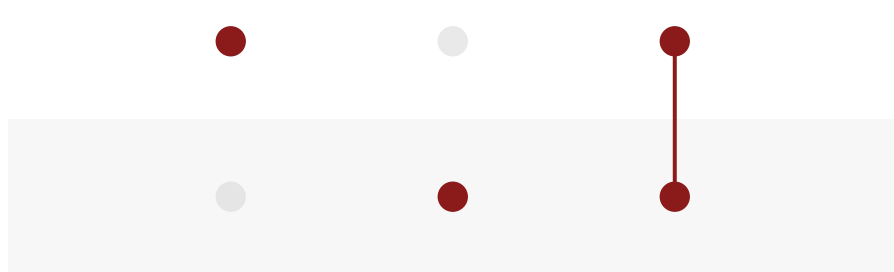

Supplement: Supplementary file 1 [file microorganisms-08-02043-s001.zip › FigS1.pdf]
